# Concrete‐Inspired Bionic Bone Glue Repairs Osteoporotic Bone Defects by Gluing and Remodeling Aging Macrophages

**DOI:** 10.1002/advs.202408044

**Published:** 2024-10-25

**Authors:** Chong Li, Wei Xu, Lei Li, Yonghui Zhou, Gang Yao, Guang Chen, Lei Xu, Ning Yang, Zhanjun Yan, Chen Zhu, Shiyuan Fang, Yusen Qiao, Jiaxiang Bai, Meng Li

**Affiliations:** ^1^ Department of Orthopedics Centre for Leading Medicine and Advanced Technologies of IHM The First Affiliated Hospital of USTC Division of Life Sciences and Medicine University of Science and Technology of China Hefei Anhui 230022 China; ^2^ Department of Orthopedics Anhui Provincial Hospital Affiliated to Anhui Medical University Hefei Anhui 230022 China; ^3^ Department of Orthopedics The Ninth People's Hospital of Suzhou Suzhou Jiangsu 215006 China; ^4^ Department of Orthopedics The First Affiliated Hospital of Soochow University 188 Shizi Road Suzhou Jiangsu 215006 China

**Keywords:** aging macrophages, biomimetic bone glue, bone defect, bone immunomodulation, osteoporotic fracture

## Abstract

Osteoporotic fractures are characterized by abnormal inflammation, deterioration of the bone microenvironment, weakened mechanical properties, and difficulties in osteogenic differentiation. The chronic inflammatory state characterized by aging macrophages leads to delayed or non‐healing of the fracture or even the formation of bone defects. The current bottleneck in clinical treatment is to achieve strong fixation of the comminuted bone fragments and effective regulation of the complex microenvironment of aging macrophages. Inspired by cement and gravel in concrete infrastructure, a biomimetic bone glue with poly(lactic‐co‐glycolic acid) microspheres is developed and levodopa/oxidized chitosan hydrogel stabilized on an organic‐inorganic framework of nanohydroxyapatite, named DOPM. DOPM is characterized via morphological and mechanical characterization techniques, in vitro experiments with bone marrow mesenchymal stromal cells, and in vivo experiments with an aged SD rat model exhibiting osteoporotic bone defects. DOPM exhibited excellent adhesion properties, good biocompatibility, and significant osteogenic differentiation. Transcriptomic analysis revealed that DOPM improved the inflammatory microenvironment by inhibiting the NF‐κB signaling pathway and promoting aging macrophage polarization toward M2 macrophages, thus significantly accelerating bone defect repair and regeneration. This biomimetic bone glue, which enhances osteointegration and reestablishes the homeostasis of aging macrophages, has potential applications in the treatment of osteoporotic bone defects.

## Graphical abstract

In this study, inspired by cement and gravel in concrete infrastructure, a biomimetic bone glue with poly(lactic‐co‐glycolic acid) microspheres (gravel) is developed and levodopa/oxidized chitosan hydrogel (cement) stabilized on an organic‐inorganic framework of nanohydroxyapatite, named **DOPM**. The bone glue is characterized via ex vivo morphological and mechanical characterization techniques, in vitro experiments with bone marrow mesenchymal stromal cells, and in vivo experiments with an aged SD rat model exhibiting osteoporotic bone defects.

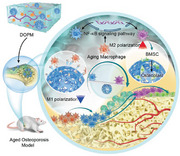

## 1 Introduction

Osteoporosis is a systemic metabolic bone disease characterized by decreased bone mass, altered bone microstructure, and increased bone fragility.^[^ ^1^ ^]^ These symptoms increase the patient's risk for fragility fractures, especially owing to minor trauma or even daily activities. Fragility fractures have a high rate of disability and mortality and can severely impact a patient's quality of life.^[^ ^2^ ^]^ These fractures are more prevalent in the middle‐aged and elderly populations.^[^ ^3^ ^]^ Surgical reduction and fixation is the common approach to treating osteoporotic fractures.^[^ ^4^ ^]^ However, comminution and screwing of the large fracture fragments may fail in fracture fixation, due to the patient's poor bone quality and slow healing.^[^ ^5^ ^]^ Furthermore, screws may cause local inflammatory reactions, while the lack of balance between mechano‐biology and mechanical stability may severely impede bone fracture healing.^[^ ^6^ ^]^ Therefore, novel implant materials are urgently required to achieve strong fracture fixation, effective fracture filling, activation of local aging macrophages, promotion of osteogenesis for osteoporotic fractures and bone defects, and good bone tissue repair and integration.^[^ ^7^ ^]^

The process of fracture filling is analogous to constructing infrastructure. Just as concrete is used in building constructions, the incorporation of a suitable adhesive material into osteoporotic bone defects may aid bone repair and regeneration.^[^ ^8^ ^]^ Concrete comprises cement, granular aggregates (gravel), and some additives and admixtures if necessary. The ratio of cement to gravel is important for its preparation.^[^ ^9^ ^]^ In this context, levodopa, with its unique catechol structure, which is the main component of mussel adhesion proteins (MAPs), exhibits excellent adhesion properties under wet conditions owing to its π‐π stacking, cation‐π bonding, hydrogen bonding, metal‐ligand bonding, and Michael addition abilities.^[^ ^10^ ^]^ We endeavored to harness the catalytic potential of N‐ethyl‐N′‐(3‐dimethylaminopropyl) carbodiimide hydrochloride (EDC) and N‐hydroxysuccinimide ester (NHS) for the formation of an amide bond between levodopa and oxidatively modified chitosan (Ocs), aiming to enhance the stability of levodopa.^[^ ^11^ ^]^ Concurrently, we innovatively proposed that the modified levodopa, exhibiting exceptional adhesion and rheological properties, could serve as a “cement” in concrete for the first time.^[^ ^12^ ^]^ The advancement in microfluidics and tissue engineering enabled the synthesis of poly(lactic‐co‐glycolic acid) (PLGA), a biodegradable polymer organic compound that has attracted significant attention in the field of biomedical engineering, owing to its good biocompatibility, nontoxicity, and superior capsule‐ and film‐forming properties.^[^ ^13^ ^]^ PLGA is widely used in the preparation of biomedical materials such as artificial catheters, slow‐release drug carriers, and tissue engineering scaffolds.^[^ ^14^ ^]^ In particular, PLGA microspheres enable the effective encapsulation and slow release of biologically active substances such as proteins and enzymes, providing a novel therapeutic strategy for bone repair.^[^ ^15^ ^]^ However, the fabrication of osteoclastic scaffolds using PLGA has not been fully explored in the literature.^[^ ^16^ ^]^ Therefore, in this study, we used PLGA microspheres, which can be called “gravel” in the context of concrete, in the synthesis of a novel biomimetic bone glue.

Given the accelerated pace of the aging process, the phenomenon of cellular senescence has emerged as a matter of paramount concern in scientific discourse.^[^ ^17^ ^]^ Aging macrophages are the most prominent immune cells in the body and may regulate their own inflammatory activation by inhibiting the cell cycle and thus reducing their phagocytosis, exacerbating aging signals.^[^ ^18^ ^]^ This aging profile is characterized by persistent cell cycle arrest and chronic low‐grade inflammation.^[^ ^19^ ^]^ In particular, in an osteoporotic bone defect microenvironment, aging macrophages exhibit a reduced ability to clear senescent or dead cells and induce the release of a large amount of inflammatory cytokines.^[^ ^20^ ^]^ These changes also skew macrophage polarization toward pro‐inflammatory M1 macrophages rather than anti‐inflammatory M2 macrophages.^[^ ^21^ ^]^ This persistent inflammatory state would adversely affect bone repair.^[^ ^22^ ^]^ This issue can be addressed by the production of a larger amount of pro‐healing M2 macrophages.^[^ ^23^ ^]^ In this context, nanohydroxyapatite (nHA), a key inorganic component of bone tissue, can precisely regulate the immune microenvironment by acting on macrophages and regulating inflammation‐related signaling pathways through paracrine mechanisms.^[^ ^24^ ^]^ However, the role of nHA in osteoporotic bone defect repair has not been fully explored thus far.^[^ ^25^ ^]^ Therefore, in this study, we fabricated metal–organic frameworks (mofs) loaded with nHA (mof@nHA) and optimized their structural design for efficient nHA loading and release.^[^ ^26^ ^]^

Inspired by the concrete analogy, in this study, we developed a novel levodopa/oxidized chitosan/PLGA/mof@nHA biomimetic bone glue to stabilize osteoporotic bone defects, promote osteogenesis, modulate the complex immune microenvironment of aging macrophages, and ultimately, facilitate bone repair. Levodopa was covalently bound to Ocs, as described previously, and PLGA was mixed with mof@nHA using a microfluidic technique to afford PLGA/mof@nHA composite microspheres with uniform size and abundant pores. These composite microspheres were then homogeneously mixed with the levodopa/oxidized chitosan glue to afford an injectable bone glue. The bone glue was evaluated on an isolated porcine bone tissue, in in vitro experiments with bone marrow mesenchymal stromal cells (BMSCs), and in in vivo experiments with Sprague–Dawley (SD) rats.

## 2 Results and Discussion

### 2.1 Synthesis and Characterization of DOPM

#### 2.1.1 Scanning Electron Microscopy and Atomic Force Microscopy

**Scheme 1:**
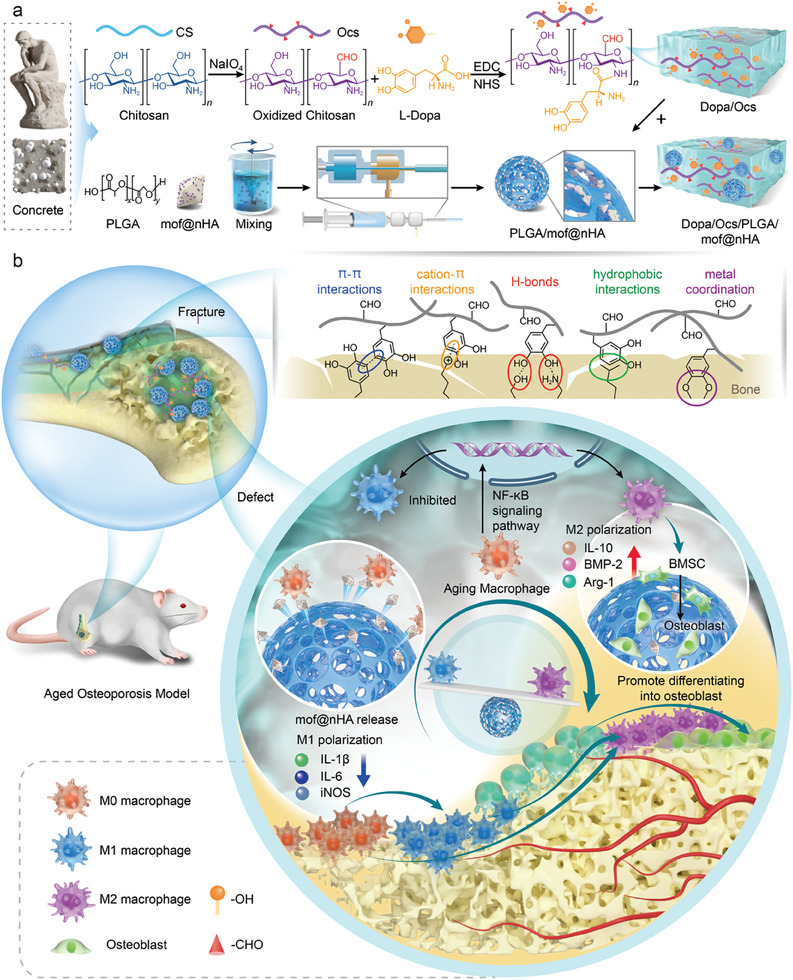
Schematic of the preparation of biomimetic bone glue inspired by concrete and its application in promoting the reconstruction of osteoporotic bone defects in the elderly. a) First, oxidized chitosan (Ocs) was prepared from chitosan and NaIO_4_. Second, levodopa (Dopa) was combined with Ocs, which was then added to a mixture of poly(lactic‐co‐glycolic acid) (PLGA) and metal–organic frameworks loaded with nanohydroxyapatite (mof@nHA) to afford levodopa/oxidized chitosan/PLGA/mof@nHA (DOPM). b) The bone glue was implanted into a femoral condylar defect model in aged osteoporotic rats. The Ocs/Dopa in DOPM immobilizes the fracture fragments and provides the necessary spatial conditions for bone healing, whereas the nHA in DOPM promotes bone regeneration, regulates the metabolism of aging macrophages, and improves the local immune microenvironment. These concurrent mechanisms can promote bone healing.

The synthetic procedure of DOPM is presented in **Scheme** **1**a. The chitosan (CS) aqueous solution is initially prepared following a specific procedure. Under dark conditions, acidic sodium periodate (NaIO_4_) is gradually added and thoroughly stirred to dissolve, facilitating the conversion of the abundant active hydroxyl groups in CS into aldehyde (‐CHO) groups. The resulting product (levodopa/oxidized chitosan) is collected using a high‐molecular‐weight filtration membrane and subsequently subjected to freeze‐drying. Following this, PLGA/mof@nHA microspheres are prepared via microfluidic technology. Finally, PLGA/mof@nHA is introduced into the levodopa/oxidized chitosan solution and mixed thoroughly (Table S1, Supporting Information).

**Figure 1:**
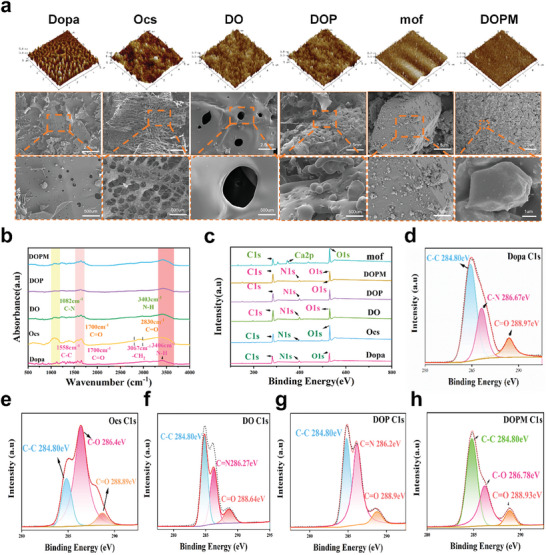
Synthesis and characterization of the DOPM bone glue. a) Atomic force microscopy and scanning electron microscopy images. b) Fourier‐transform infrared spectra. c) X‐ray photoelectron (XPS) survey spectra. (d‐h) XPS spectra of Dopa, Ocs, DO, DOP, and DOPM (n = 6; data shown represent mean ± SD; *p < 0.05, **p < 0.01, ***p < 0.001, ns, no significance).

The microstructure of the biomimetic bone glue is crucial, as it will be in direct contact with the bone tissue. Hence, the surface morphology of DOPM was studied using atomic force microscopy (AFM) and scanning electron microscopy (SEM) at high magnifications (**Figure** **1**a). The root‐mean‐square roughness (*R*_q_) and average roughness (*R*_a_) of Dopa measured by AFM were 1.27 and 0.993 nm, respectively, with its roughness reaching 10.8 nm. In addition, the *R*_q_ and R_a_ of the Ocs were 5.12 and 3.74 nm, respectively, with its roughness reaching 78.6 nm. Dopa/Ocs exhibited smaller *R*_q_ and larger R_a_ than those of Dopa and Ocs (Table S2, Supporting Information), indicating that the reaction between the amino groups of Dopa and the carboxyl groups of Ocs reduced the overall surface roughness of Dopa/Ocs.^[^ ^27^ ^]^ The R_a_ of DOPM increased by 3% and its *R*_q_ increased by 16% compared to those of DOP. Hence, the roughness values of DOPM are in a satisfactory range. SEM images of Ocs revealed homogeneous and dense pores with a pore size of 120 ± 14 µm, which is conducive to its adherence to the bone surface and provides a good base condition for bone healing. Furthermore, the SEM images of the DOP surface reveal a large number of densely packed microspheres. While the SEM image of mof@nHA demonstrated a large number of nanohydroxyapatite molecules with their typical hexagonal structure, the DOPM group exhibited tightly bound components in a concrete‐like structure.^[^ ^28^ ^]^ These results show that the new biomimetic bone glue has satisfactory morphological properties.

#### 2.1.2 Fourier‐Transform Infrared Spectroscopy and X‐Ray Photoelectron Spectroscopy

The chemical bonding in Dopa, Ocs, PLGA, and mof@nHA was investigated via Fourier‐transform infrared spectroscopy (FTIR). Dopa exhibited peaks at 3406, 1700, and 3000–3100 cm^−1^, corresponding to N–H, C ═ O, and aromatic C–H stretching vibrations, respectively. Ocs, which contains –CHO groups obtained from the oxidation of the –OH groups of CS, exhibited a characteristic –CHO peak at 2830 cm^−1^. The Dopa/Ocs spectrum showed smaller peak intensities at 1000–1200 and 1500–1700 cm^−1^ compared to those in Dopa and Ocs, respectively, which could be attributed to the formation of peptidic bonds (–CO–NH–). These results confirm the stable chemical bonding in DOPM (Figure 1b). Figure 1c shows the XPS survey spectra of Dopa, Ocs, DO, DOP, and DOPM, while Figure 1d–h show the XPS spectra of all the raw materials and DOPM. The O content slightly decreased, whereas the C content slightly increased in Dopa/Ocs compared to those in Dopa and Ocs. This confirms peptide bond formation via the dehydration condensation reaction.

#### 2.1.3 Rheological Analysis

**Figure 2:**
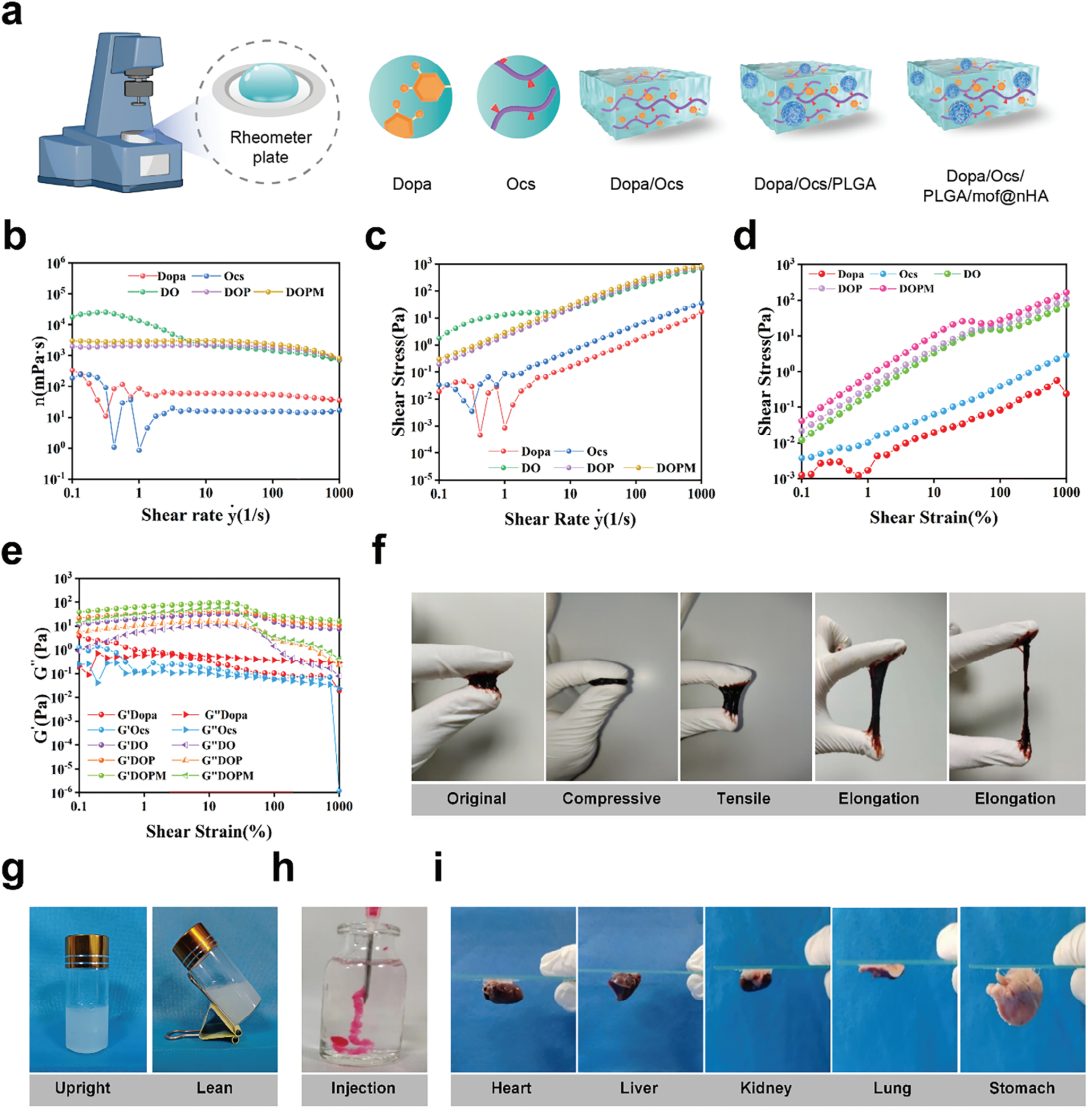
Rheological and bioadhesion analysis of DOPM bone glue. a) Flow chart of the rheological experiments. b) Kinetic viscosity tests to evaluate the flow properties of DOPM. c,d) Shear stress tests to determine the response of DOPM to different shear rates. e) Amplitude test at a fixed frequency to assess the viscoelastic properties of DOPM. f) Compression and tensile toughness tests to measure the mechanical strength and durability of DOPM. g) Solidification tests to investigate the curing behavior and setting time of DOPM. h) Injectability of DOPM to assess its practical application in medicine. i) Bioadhesion experiments to evaluate the adhesive strength of DOPM toward various tissues (heart, liver, kidney, lung, and stomach) and a glass plate (n = 6; data shown represent mean ± SD; *p < 0.05, **p < 0.01, ***p < 0.001, ns, no significance).

Shear thinning behavior and elastic modulus analyses were conducted to study the rheological behavior of DOPM.^[^ ^29^ ^]^ The viscosity measurements and fixation were performed in the flat plate measurement mode (**Figure** **2**a). The shear thinning behavior of DOPM was quantified by fitting the viscosity profile to a power law equation. Dopa/Ocs exhibited a higher viscosity than DO, DOP, and DOPM at shear rates of 0.1–1000 s^−1^ but lower viscosities at a shear rate of 7.5 s^−1^, which corresponds to an increase in stress (Figure 2b). Notably, DOPM demonstrated a maximum stress of 164.96 ± 5.23 Pa at a shear rate of 1000 s^−1^ and the highest viscosity among the other raw materials (Figure 2c,d). The viscoelastic behavior of DOPM was examined via an amplitude sweep at a fixed frequency (Figure 2e). The energy storage modulus (G′) of a material reflects its resistance to compressive deformation and the mechanical strength of the analogous solidity. The loss modulus (G″) reflects the energy dissipated by the material during stress. Bone resins with sufficient mechanical strength and yield stress exhibit excellent self‐supporting behavior. Dopa/Ocs exhibited higher G′ and G″ values than those of Dopa and Ocs. However, all raw materials and DOPM have higher G′ values than the G″ values, which confirm the stable structure of the DOPM bone glue. DOPM exhibited the highest G′ among all the analyzed samples, indicating its high adhesion strength.^[^ ^30^ ^]^ Compression and tensile tests demonstrated the excellent flexibility of the DOPM bone glue (Figure 2f), while injection and adhesion experiments in rat organs and glass showed that the application of bone glue was flexible and practically maneuverable (Figure 2g–i).

### 2.2 Mechanical Properties and In Vitro Adhesion Strength of DOPM

**Figure 3:**
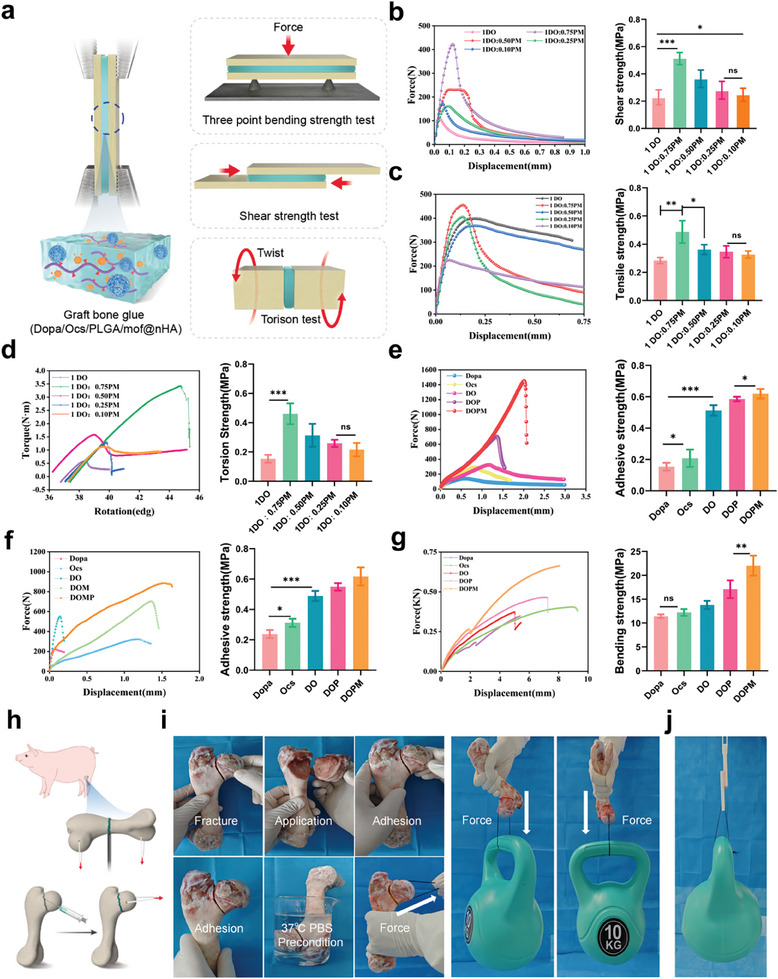
Mechanical properties and ex vivo adhesion experiments on isolated porcine bone bonding experiments with the DOPM bone glue. a) Mechanical tests conducted using an electronic universal testing machine. b) Shear tests of DOPM mixtures with different glue‐to‐spheres ratios to assess their adhesive strengths. c) Tensile tests of DOPM mixtures with different glue‐to‐spheres ratios to determine tensile forces. d) Torsion experiments of DOPM mixtures with different glue‐to‐spheres ratios to study the response of the bone glue to torsional stress. e) Shear strengths of bone glues with optimal glue‐to‐spheres ratios determined based on experimental results. f) Tensile strengths of bone glues with optimal glue‐to‐spheres ratios determined based on experimental results. g) Bending strengths of bone glues with optimal glue‐to‐spheres ratios determined based on experimental results. h,i) Adhesive bonding performance of DOPM in isolated porcine femoral head osteotomy repair experiments. j) Shear mechanical properties of porcine bone slices to assess the bonding strength of DOPM (n = 6; data shown represent mean ± SD; *p < 0.05, **p < 0.01, ***p < 0.001, ns, no significance).

The mechanical properties of the raw materials and DOPM were tested through shear, tensile, torsion, and bending tests (**Figure** **3**a).^[^ ^31^ ^]^ Dopa/Ocs is equivalent to the cement in concrete, whereas PLGA/mof@nHA is equivalent to the gravel. Hence, we determined the optimal cement/gravel (i.e., Dopa/Ocs: PLGA/mof@nHA) ratio that affords an ultrahigh adhesion bonding effect. The following five mixtures of Dopa/Ocs:PLGA/mof@nHA were prepared: 1 g DO, 1 g DO:0.75 g PM, 1 g DO:0.50 g PM, 1 g DO:0.25 g PM, and 1 g DO:0.10 g PM. These mixtures were subjected to shear, tensile, and torsion tests, and the corresponding scatter error histograms were plotted (Figure 3b–d). Among the mixtures, 1 g DO:0.75 g PM showed the highest shear strength (0.5110  ± 0.18 MPa), tensile strength (0.4876  ± 0.087 MPa), and torsional strength (0.4606  ± 0.045 MPa). Thus, this optimal mass ratio was selected for further shear, tensile, and three‐point bending tests, and the corresponding displacement‐force curves and adhesion strength histograms were plotted. The shear stress of DOPM was 1450 N, and its shear strength was 0.6196 ± 0.058 MPa (Figure 3e). This was considered optimal because it was deemed to be the best value among all experimental groups. DOPM exhibited a tensile force of 866 N at 1.62 mm and a tensile strength of 0.6171 ± 0.023 MPa (Figure 3f). The three‐point bending test analyzes the bending compressive tolerance of materials. Even though Dopa and Ocs have comparable bending strengths, DOPM showed a high bending strength of 22.07 ± 2.67 MPa (Figure 3g).

For *ex vivo* adhesion experiments, the femoral head of an isolated pig femur was sawed off and the DOPM bone glue was evenly applied to the broken end to fix the fracture, followed by rejoining of the broken ends.^[^ ^32^ ^]^ The resultant femur was then immersed in a PBS solution and allowed to stand overnight at 37 °C. During the test, the femoral head successfully lifted a 10 kg weight without breaking (Figure 3h,i). Furthermore, DOPM was employed to bond the ends of the excised porcine bone fragments. The bone fragments, with an adhesion area of 5 cm^2^, successfully supported 10‐kg kettlebells (Figure 3j), demonstrating an adhesion strength of 196.2 kPa.

### 2.3 Evaluation of In Vitro and Ex Vivo Compatibility and Sustainable Degradability of DOPM

**Figure 4:**
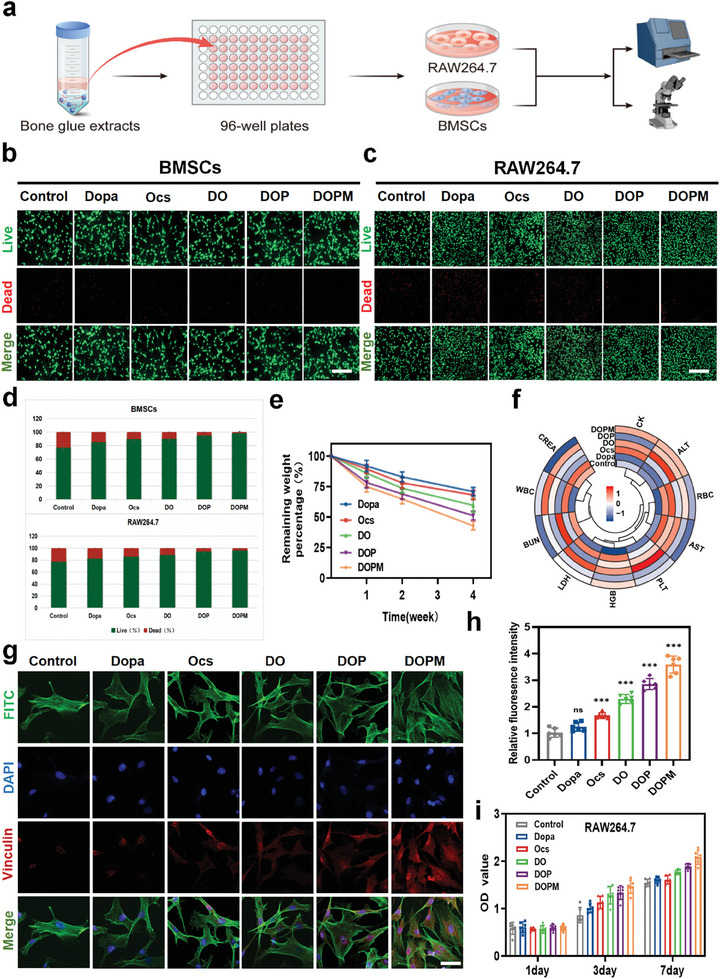
Cytobiocompatibility of the DOPM bone glue. a) Schematic of the cellular experiments performed to assess the cytobiocompatibility of DOPM bone glue. b,c) Live/dead staining of RAW264.7 cells and BMSCs after 72 h of cultures with Dopa, Ocs, DO, DOP, or DOPM to assess cell viability (scale bar = 100 µm). d) Semi‐quantitative analysis of live/dead staining to determine the percentage of viable cells. e) Mass loss percentages of Dopa, Ocs, DO, DOP, and DOPM after 1, 2, and 4 weeks of subcutaneous implantation to assess its biodegradability. f) Heat map of blood parameters in SD rats 4 weeks after subcutaneous implantation to evaluate the systemic effects of DOPM. g) Vinculin immunofluorescence staining of BMSCs 72 h after cultures with Dopa, Ocs, DO, DOP, DOPM to determine cellular adhesion and morphology (scale bar = 20 µm). h) Semi‐quantitative analysis of vinculin immunofluorescence staining relative to the control group (PBS) to quantify cellular adhesion strength. i) Viability of RAW264.7 cells after 1, 3, and 7 d of culture with control group (PBS), Dopa, Ocs, DO, DOP, or DOPM using the CCK‐8 kit method (n = 6, data shown represent mean ± SD; *p < 0.05, **p < 0.01, ***p < 0.001, ns, no significance).

An excellent biocompatibility and in vivo non‐toxic degradation are the prerequisites for the applications of bone glue. Hence, the biocompatibility of BMSCs and RAW264.7 cells was determined (**Figure** **4**a). The cells were inoculated with Dopa, Ocs, DO, DOP, and DOPM for 72 h, stained using the live/dead staining kit, and analyzed using an inverted fluorescence microscope. PBS was used as the blank (control group). The number of dead cells in the DO, DOP, and DOPM groups was significantly smaller than that in the blank (Figure 4b,c). The percentage bar graph analysis clarified the live/dead cell ratios in the in vitro cell cultures (Figure 4d). As the affinity of the bone glue for BMSCs is crucial for promoting bone defect healing, we studied cultured BMSCs (for 7 d) via laser confocal microscopy to observe the expression of the adhesion spot protein (Vinculin).^[^ ^33^ ^]^ In the case of the control group, the filamentous feet of BMSCs were short and thin, the cells were more dispersed from each other, and the protein fluorescence intensity was weak. In contrast, BMSCs cultured with DOPM were aggregated, and their protein fluorescence intensity was significantly higher than those of Dopa, Ocs, and the control group (Figure 4g,h). This could be attributed to the production of a large number of cytokines, such as transforming growth factor‐β (TGF‐β) and bone morphogenetic protein (BMP), which regulate the internal environment and promote cell growth. This suggests that the DOPM bone glue promotes bone repair in BMSCs. Next, BMSCs cell proliferation was investigated. The cells were cultured for 1, 3, and 7 d, and their OD values were measured using a CCK‐8 kit to confirm their cell proliferative activity (Figure 4I; Figure S2, Supporting Information).

The in vivo degradation of DOPM was studied. SD rats were subcutaneously implanted with ≈100 µL of lyophilized Dopa, Ocs, DO, DOP, or DOPM and randomly divided into five groups.^[^ ^34^ ^]^ After 1, 2, and 4 weeks, the implantation sites were opened, and the weight of the remaining material was measured (Figure S1a,b, Supporting Information). Only small amounts of DOP and DOPM remained, and the surrounding tissues showed obvious swelling and contracture. However, a larger amount of DO remained, and clearer boundaries were observed between DO and the surrounding tissue. The line graphs of the remaining weights of DO, DOP, and DOPM clearly showed their degradation trends (Figure 4e). The skin at the implantation site was analyzed by hematoxylin and eosin (H&E) staining. No significant inflammatory cell infiltration was observed in DO or DOP. After degradation of the material, the skin tissue surrounding DOPM was not significantly different from that of normal skin (Figure S1c, Supporting Information).

To verify whether the subcutaneous implantation had any negative effects on the SD rats, blood specimens were collected and analyzed after 4 weeks (Figure 4f). Routine physical and chemical tests of their blood showed normal ranges for red blood cell counts, hemoglobin, white blood cells, and platelets. As the liver and kidney are the main metabolic organs of rats, we performed alanine aminotransferase, alanine transaminase, creatinine, blood urea nitrogen, lactate dehydrogenase, and creatine kinase analyses.^[^ ^35^ ^]^ These indices did not show any significant changes after 4 weeks of implantation. All the abovementioned results confirm the non‐toxicity and biocompatibility of the DOPM bone glue.

### 2.4 In Vitro Osteogenesis Promotion Experiments with DOPM Bone Glue

**Figure 5:**
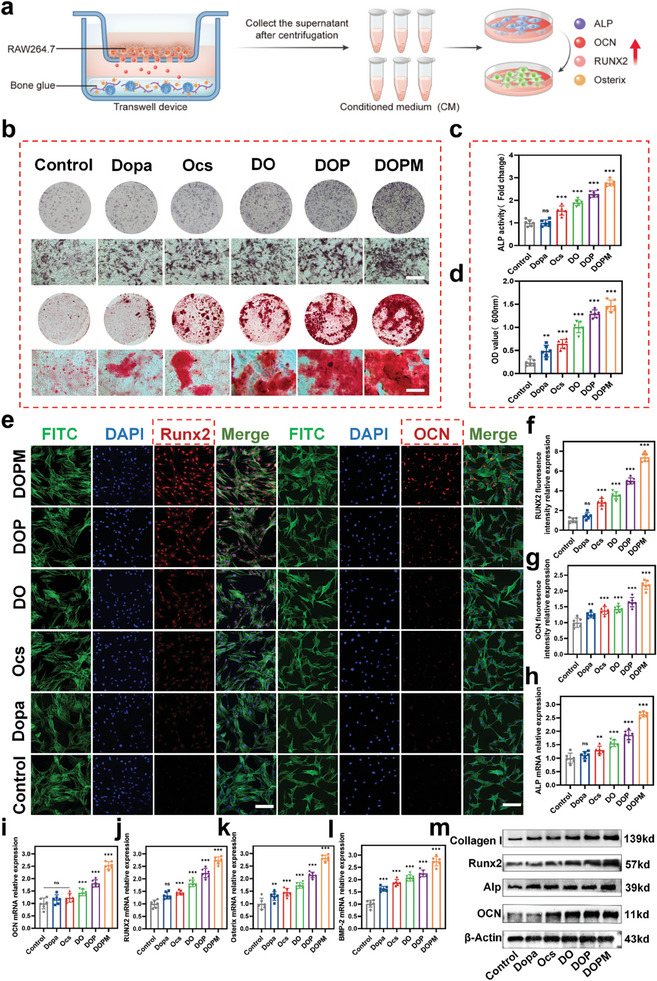
In vitro experiments of BMSCs cultured with the DOPM bone glue to assess osteogenic differentiation. a) Schematic of the experimental setup for evaluating in vitro osteogenic differentiation induced by DOPM. b) Alkaline phosphatase (ALP) and alizarin red (ARS) staining of BMSCs cultured with DOPM and its raw materials (scale bar = 200 µm). c,d) Semi‐quantitative analysis of ALP and ARS staining to quantify osteogenesis. e) Immunofluorescence staining images showing osteogenic differentiation markers (RUNX2 and OCN) in BMSCs (scale bar = 20 µm). f,g) Semi‐quantitative analysis of the immunofluorescence intensities of RUNX2 and OCN to assess their expression levels. h–l) Gene expression levels of ALP, OCN, RUNX2, Osterix, and BMP‐2 using RT‐PCR. m) Protein imprinting (Western blot) experiments with Col‐I, RUNX2, ALP, and OCN to confirm their protein (n = 6; data shown represent mean ± SD; *p < 0.05, **p < 0.01, ***p < 0.001, ns, no significance).

Osteoporosis involves the destruction and thinning of bone trabeculae, reduced bone density, increased bone marrow fat, changes in the bone microstructure, and changes in the composition of the bone matrix, all of which result in a significant reduction in the osteogenic capacity of the local bone tissue.^[^ ^36^ ^]^ Osteogenic differentiation is a process in which BMSCs form osteoblasts, a crucial step for bone tissue repair.^[^ ^37^ ^]^ Hence, we analyzed the osteogenic differentiation in BMSCs cultured with DOPM and its raw materials to promote bone tissue healing. The control group was treated with PBS (**Figure** **5**a). After 7 days of cell culturing, the samples were analyzed via alkaline phosphatase (ALP) staining (Figure 5b). DO, DOP, and DOPM were deeply stained, whereas the control group, Dopa, and Ocs groups were lightly stained, as observed by the naked eye. These results were confirmed via microscope observations. The ALP activity was quantified using an alkaline phosphatase activity assay kit. DOPM exhibited the highest ALP activity (Figure 5c), along with a large number of aggregated red mineralized calcium nodules on day 21 (Figure 5b). DOP and DOPM demonstrated higher alizarin red (ARS) staining than the control group, Dopa, and Ocs (Figure 5d). The ALP and ARS staining results confirmed that BMSCs cultured with DOPM underwent osteogenesis and synthesized a large amount of alkaline phosphatase to regulate the mineralization of the bone matrix.^[^ ^38^ ^]^

Moreover, two osteogenesis‐related genes, Runt‐related transcription factor 2 (RUNX2) and osteocalcin (OCN), were labeled using immunofluorescence staining. The fluorescence expression of BMSCs cultured with DOPM was higher than that with DOP. However, a weak fluorescence was observed in the control group (Figure 5e). Subsequently, their fluorescence intensities were quantified (Figure 5f,g). The osteogenic activity of DOPM was further assessed using the reverse transcription polymerase chain reaction (RT‐PCR). The mRNA expression of five osteogenesis‐related genes (ALP, OCN, RUNX2, Osterix, and BMP‐2) was investigated.^[^ ^39^ ^]^ The cell proliferation in the presence of DOPM was high (Figure 5h,i). Western blot experiments were performed to identify the following osteogenesis‐related proteins: collagen type I (Col‐I), RUNX2, ALP, and OCN (Figure 5m). Their relative protein expression was also quantified (Figure S3, Supporting Information). Thus, the protein imprinting results were consistent with those of PCR and immunofluorescence staining. These results confirm the excellent osteogenic differentiation ability of the DOPM bone glue in in vitro cell cultures.

### 2.5 In Vitro Phenotypic Transformation of Aging Macrophages in the Presence of DOPM to Re‐Establish the Immune Microenvironment

**Figure 6:**
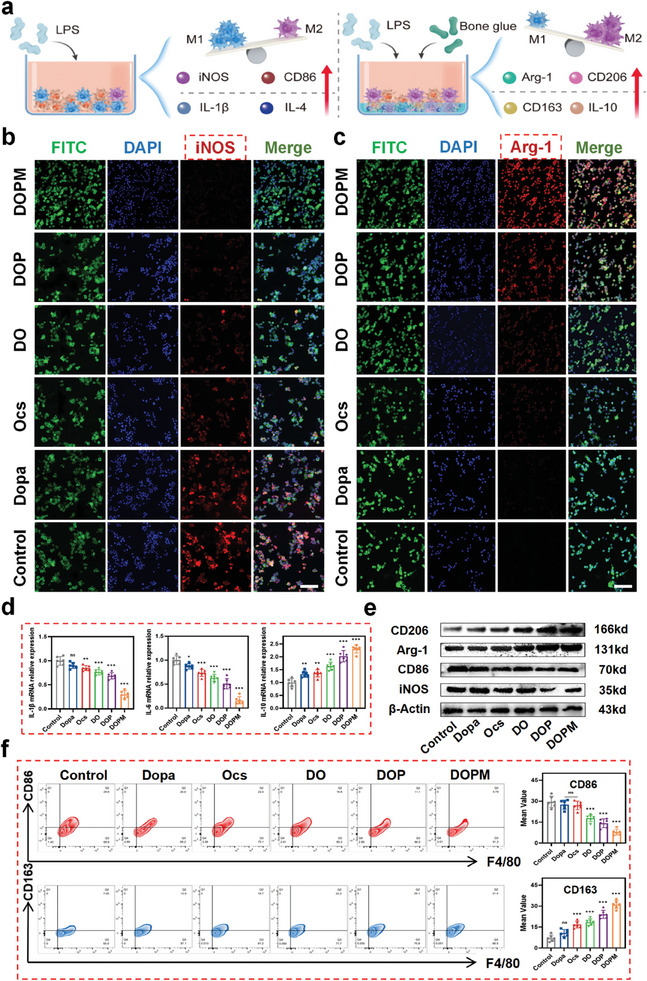
Immunomodulatory effects of the DOPM bone glue on the immune microenvironment around aging macrophages of the bone tissue. a) Schematic of the experimental setup for assessing the in vitro modulatory effects of DOPM on aging macrophages. b,c) Immunofluorescence staining images of RAW264.7 cells treated with DOPM to visualize phenotypic changes (scale bar = 20 µm). d) Real‐time quantitative reverse transcription polymerase chain reaction (RT‐PCR) to determine the gene expression levels of IL‐1β, IL‐6, and IL‐10 in cultured RAW264.7 cells. e) Western blot experiments with RAW264.7 cells to assess the protein expression of iNOS, CD86, Arg‐1, and CD206. f) Flow cytometry and quantitative analysis to evaluate the phenotypic and functional shifts in RAW264.7 cells (n = 6; data shown represent mean ± SD; *p < 0.05, **p < 0.01, ***p < 0.001, ns, no significance).

Aging macrophages are more likely to polarize to the M1 phenotype in the immune microenvironment of osteoporotic bone defects, increasing the release of pro‐inflammatory cytokines to cause high inflammation, which prevents bone tissue repair.^[^ ^40^ ^]^ Previous studies have demonstrated that certain chemicals, such as lipopolysaccharides (LPS), can simulate the senescent phenotype of macrophages.^[^ ^41^ ^]^ Hence, we utilized LPS (1 µg mL^−1^) to simulate the inflammatory microenvironment of aging macrophages and supplemented it with DOPM. Subsequently, we investigated the effect of DOPM on macrophage polarization and the associated inflammatory factors (**Figure** **6**a). The M1 phenotypic marker nitric oxide synthase (iNOS) and the M2 ideal phenotypic indicator arginase 1 (Arg‐1) were visualized via immunofluorescence staining. The fluorescence intensity of iNOS in the control group was significantly higher than that in the presence of DO, DOP, and DOPM (Figure 6b). In contrast, the DOPM‐treated sample showed the weakest fluorescence intensity for iNOS but a strong fluorescence intensity for Arg‐1 (Figure 6c). The abovementioned fluorescence intensities were quantified (Figure S4, Supporting Information). The aging macrophages cultured with LPS and DOPM underwent macrophage polarization to the M2 state. Furthermore, the mRNA and protein expression of the DOPM‐treated macrophages were investigated via RT‐PCR and western blotting experiments. The mRNA expression of the pro‐inflammatory factors such as IL‐1β and IL‐6 was higher in the control group compared to those of DOPM and other raw materials. However, the mRNA expression of the anti‐inflammatory factor IL‐10 was higher in the DOPM‐treated cells (Figure 6d). Meanwhile, the expression level of the iNOS gene, a marker for M1 macrophages, was downregulated, while the expression level of the Arg‐1 gene was upregulated. The western blotting results were consistent with the RT‐PCR results (Figure S5, Supporting Information). The protein expression of the M2‐type markers CD206 and Arg‐1 was significantly higher in the presence of DOPM than those of the other materials (Figure 6e; Figure S6, Supporting Information).

To verify whether the DOPM‐treated aging macrophages were of the M2 type, we evaluated the CD163/F4/80 and CD86/F4/80 expression ratios via flow cytometry (Figure 6f).^[^ ^42^ ^]^ The CD163/F4/80 ratio in the presence of mof@nHA was 113% higher than that in the absence of mof@nHA and 428% higher than that of the control group. The CD163/F4/80 ratio also increased in the presence of DOP and DOPM. The CD86/F4/80 ratio was significantly lower in the presence of DOPM than in the presence of its raw materials. This suggests that the DOPM bone glue demonstrated significant immunomodulatory effects in the in vitro experiments, which involved promoting the polarization of aging macrophages toward the M2 type, exerting anti‐inflammatory effects, inhibiting the secretion of pro‐inflammatory factors, and enhancing the production of anti‐inflammatory molecules. These combined effects effectively improved the immune microenvironment around the osteoporotic bone defects.

### 2.6 Transcriptome Sequencing to Elucidate the Immunoregulatory Mechanism of DOPM

**Figure 7:**
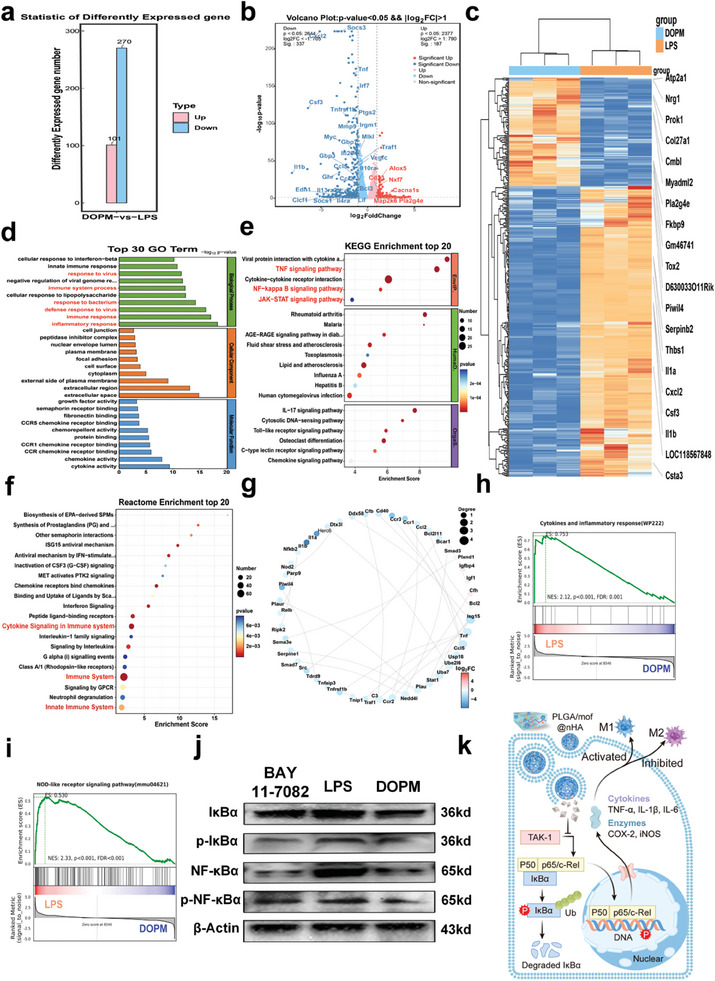
Transcriptome sequencing analysis of the immunoregulatory mechanisms of DOPM bone glue around aging macrophages. a) Differential gene expression analysis to identify the genes affected by DOPM. b,c) Volcano plots and heat maps to visualize the changes in the inflammation‐related gene expression. d–f) Gene Ontology, Kyoto Encyclopedia of Genes and Genomes, and Reactome enrichment analyses to determine the biological pathways and mechanisms that are influenced by DOPM. g) Protein‐protein interaction network analysis to understand the complex interactions between differentially expressed proteins. h,i) Gene set enrichment analysis to investigate the enrichment of gene sets associated with inflammatory responses and the activation of nucleotide oligomerization domain (NOD)‐like receptor signaling pathways. j) Western blot experiments to validate the activation of key proteins in the NF‐κB signaling pathway. k) Schematic of the regulatory effect of DOPM on the NF‐κB signaling transduction pathway (n = 6; data shown represent mean ± SD; *p < 0.05, **p < 0.01, ***p < 0.001, ns, no significance).

Transcriptome sequencing can reveal the molecular mechanisms involved in biological pathways and in the development of diseases. RNA‐Seq transcriptome sequencing technology has become an indispensable tool for analyzing differential gene expression/mRNA variable splicing at the transcriptome level. Furthermore, methods such as immunofluorescence revealed the participation of osteoclasts in regulating the metabolism of aging macrophages to inhibit their inflammatory response. However, the biological events during this metabolism remain unclear. Therefore, we performed transcriptome sequencing on LPS and DOPM‐treated macrophages.^[^ ^43^ ^]^ Principal component analysis (PCA) and correlation analysis indicated good sample reproducibility for all samples, however, significant variations were observed between the samples, with a PC1 of 85.85% (Figures S7 and S8, Supporting Information). A high level of confidence was observed for the results. Differential gene screening of DOPM‐treated RAW264.7 cells revealed 101 up‐regulated genes and 270 down‐regulated genes (**Figure** **7**a). Volcano maps clarified the overall distribution of the inflammatory gene markers in RAW264.7 cells. A large number of inflammatory response‐related factors, such as Il1b, Lif, Mlkl, and Mmp9, were more suppressed in the presence of DOPM than with LPS (Figure 7b). Heat maps for gene expression can help visualize the data distribution and changes in differential gene expression (Figure 7c). Subsequently, gene ontology enrichment analysis was performed on the differentially expressed genes to characterize their functions, mainly focusing on inflammation and immune evaluation. As expected, genes associated with bacterial and viral inflammation as well as immune responses were significantly expressed (Figure 7d; Figure S9, Supporting Information). Next, a KEGG pathway analysis was performed on the differential protein‐coding genes (Figure 7e). The inflammatory expression‐related signaling pathways, such as the NF‐κB and TNF signaling pathways, were inhibited after DOPM treatment. These results were corroborated via reactome enrichment analysis (Figure 7f). A gene‐gene interaction network analysis of DEG in the NF‐κB pathway showed significant downregulation of the NF‐κB‐associated major inflammatory cytokine signaling (Figure 7g). Gene set enrichment analysis confirmed the activation of cytokine and inflammatory responses in the presence of LPS and the inhibition of the NF‐κB signaling pathway in the presence of DOPM (Figure 7h,i). Although multiple genes and signaling pathways were involved in the inflammatory response, we assumed that DOPM may have exerted an anti‐inflammatory effect through the NF‐κB signaling pathway.^[^ ^44^ ^]^ To verify our assumption, we evaluated the expression of the p65 and IκBα proteins associated with the NF‐κB signaling pathway via western blotting experiments. The phosphorylation levels of p65 and IκBα were lower in the presence of DOPM than those with LPS. Moreover, the expression levels of BAY 11–7082, an NF‐κB inhibitor, were comparable to those of DOPM (Figure 7j). This suggests that DOPM effectively inhibits the phosphorylation activity of key proteins in the NF‐κB signaling pathway, thus inhibiting the inflammatory effects of aging macrophages (Figure 7k).

### 2.7 Role of DOPM in the Repair and Reconstruction of Osteoporotic Bone Defects in Aged Osteoporotic Rats

**Figure 8:**
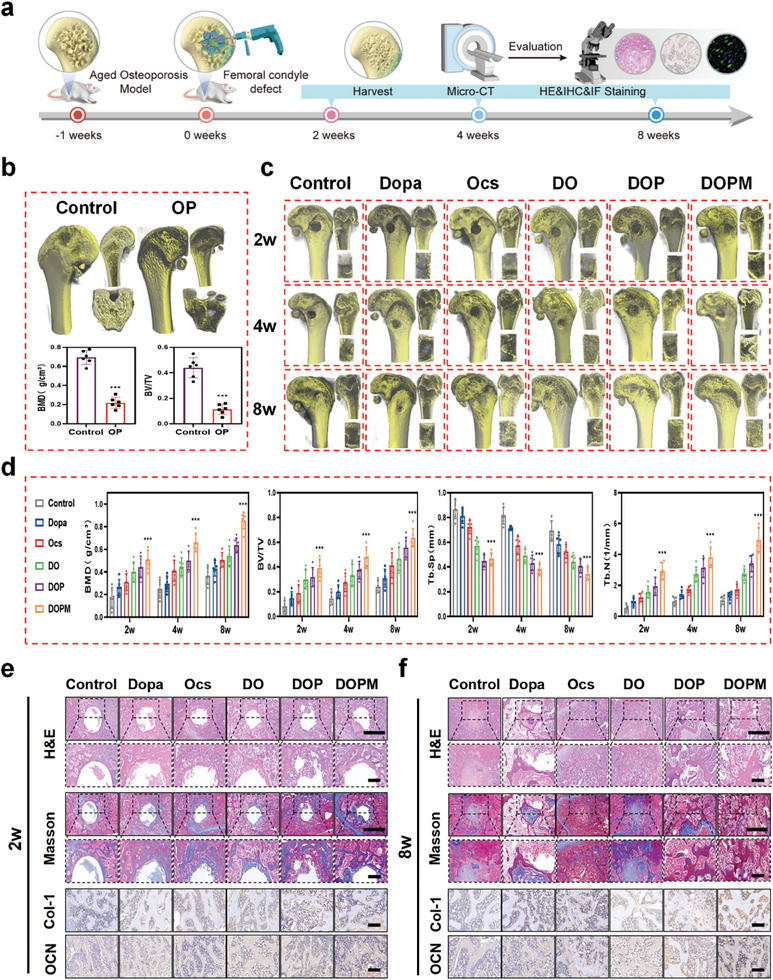
Evaluation of the in vivo efficacy of the DOPM bone glue in promoting the repair of osteoporotic bone defects in elderly rats. a) Animal experimental design and timeline. b) Aged osteoporosis model that simulates osteoporotic conditions in the elderly population. c,d) Micro‐CT 3D reconstruction and quantitative analysis on femoral specimens to evaluate bone repair and regeneration. e,f) Histological analysis with hematoxylin and eosin (H&E) and Masson staining [scale bars: 50 µm (top) and 20 µm (bottom)] and immunohistochemical fluorescence staining for collagen type I (Col‐1) and osteocalcin (OCN) at bone defect sites after 2, 4, and 8 weeks following DOPM implantation (scale bar = 20 µm) (n = 6; data shown represent mean ± SD; *p < 0.05, **p < 0.01, ***p < 0.001, ns, no significance).

A schematic of the in vivo experimental procedure is shown in **Figure** **8**a. The femur specimens of two‐ and twenty‐one‐month‐old SD rats were collected and quantitatively analyzed via micro‐CT reconstruction.^[^ ^45^ ^]^ Their bone mineral density (BMD) was only 28% of that of the two‐month‐old SD rats, and the relative bone volume fraction (bone volume/group volume; BV/TV) decreased by 76% from that of the control group (Figure 8b). The number of trabeculae (Tb.N) and trabecular thickness (Tb.Th) were significantly lower than those of the control group (Figure S10, Supporting Information). Such results confirm the successful realization of the aged osteoporosis model. A model of a femoral condylar bone defect was established using the validated aged osteoporosis model (Figure S11, Supporting Information).

The bone‐healing effects of DOPM and its raw materials were investigated by implanting them into the bone defect model. The control group was implanted with PBS. Different femur groups were removed at 2, 4, and 8 weeks from the bone defect model and reconstructed into 3D structures using micro‐CT for histological analysis. The internal organs (heart, liver, spleen, kidney, and lungs) of the SD rats were collected 8 weeks after implantation of DOPM for toxicity assessment. H&E staining of these internal organs did not reveal any obvious pathological changes (Figure S12, Supporting Information), indicating the nontoxicity of DOPM in the SD rats. Micro‐CT was used to visualize the microscopic changes of bone regeneration (Figure 8c). Even though the bone tissue increased with time in the presence of DO, DOP, and DOPM, the bone repair during the same period was different for each of them. While the bone defects were completely covered by connective tissue in the presence of DOPM by Week 2, they were completely covered by bone‐like tissue by Week 8. The control exhibited only partial bone‐tissue filling by Week 8.

Quantitative analysis showed that DOP was more osteogenic than DO over the fixation period (Figure 8d), which was attributed to the scaffolding provided by PLGA during the repair process. The highest bone analysis parameters were achieved for the model implanted with DOPM, which is attributed to the better osteoconductivity and osteoinductivity of the PLGA/mof@nHA microspheres.^[^ ^46^ ^]^ By Week 8, the BMD, BV/TV, and Tb.N of the DOPM group were 262%, 289%, and 418% that of the control group, respectively. Such results confirm the bone repairing effect of DOPM in aged osteoporosis rats. Furthermore, the DOPM‐treated samples collected at 2, 4, and 8 weeks were subjected to histological and immunohistochemical staining. The H&E and Masson staining images of the femoral condylar bone defects after the different treatments are shown in Figure 8e,f and Figure S13 (Supporting Information). H&E staining aids in visualizing general bone tissue structures and cells, while Masson staining helps in visualizing connective tissue. In these staining tests, a collagen fiber network appears clear, whereas the newly‐formed bone is stained blue, and the mature bone is stained red. The amount of new bone formation in the control group was lower than those of DOPM and its raw materials at different time intervals. The amount of bone formation in the DOPM sample was higher than those of the other groups. Masson staining showed different amounts of collagen fibers at different time periods. The DOPM group exhibited the highest percentage of mature bone tissues, indicating the best osteogenic effect. To assess the potential differences in osteogenic gene expression during bone defect repair, the specimens were subjected to an immunohistochemical staining assay for osteogenesis‐related genes. The highest expression of OCN and Col‐I was observed in osteoporotic bone defects treated with DOPM for different periods.

**Figure 9:**
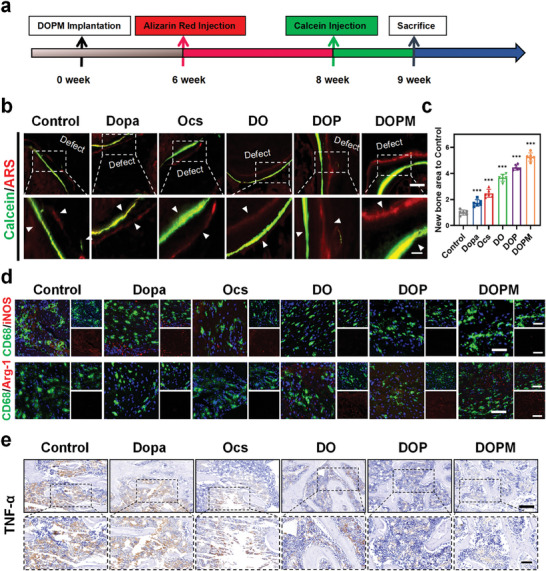
In vivo assessment of neonatal bone staining and regulation of the metabolic effects of aging macrophages in the DOPM bone glue. a) Schematic of the timing and the sequence of the stain injections used in the neonatal bone staining experiments. b,c) Fluorescent labeling (red, Alizarin; green, Calcein) and quantitative analysis on neonatal bone tissue to assess bone formation and mineralization. d) Immunofluorescence staining to identify aging macrophages in local tissues using CD68 (green), iNOS, and Arg‐1 (red) markers. e) Immunohistochemical analysis for TNF‐α pro‐inflammatory markers to evaluate inflammatory responses [scale bars: 50 µm (top) and 20 µm (bottom)] (n = 6; data shown represent mean ± SD; *p < 0.05, **p < 0.01, ***p < 0.001, ns, no significance).

Bone regeneration is accompanied by mineral aggregation. Hence, to further understand the spatiotemporal behavior of bone formation after the implantation of DOPM, alizarin red and calcein yellow chlorophyll were intramuscularly injected at a certain time interval as continuous osteogenic fluorescent markers (**Figure** **9**a).^[^ ^47^ ^]^ After the implantation of DOPM in the SD rats, a wide range of intense red and green fluorescence was observed around the new bone (Figure 9b). Such a finding indicates that DOPM significantly promotes early and late osteogenesis in osteoporotic bone defects. In contrast, the control group showed weak and scattered fluorescence, suggesting slower mineral deposition in osteoporotic bone defects, leading to difficulties in bone healing. Quantitative analysis showed that DOPM‐treated bone defects exhibited the largest area of new bone mineralization (Figure 9c). As bone tissue repair involves the immune microenvironment of the defective region, bone tissue samples were analyzed via immunofluorescence and immunohistochemical staining for inflammatory factors and to determine the macrophage phenotype (Figure 9d). The macrophages were labeled using CD68 (green fluorescence), iNOS (red fluorescence), and Arg‐1 (red fluorescence) to represent specific M1 and M2 macrophage phenotypes. DOPM‐implanted tissues demonstrated the weakest iNOS fluorescence and the strongest Arg‐1 fluorescence, suggesting that DOPM significantly promotes macrophage polarization toward M2, thus promoting the reconstruction of bone defects. Immunohistochemical staining of TNF‐α, a pro‐inflammatory marker, showed a lower number of inflammatory markers in the DOPM‐implanted tissues than that with other materials (Figure 9e). Furthermore, the DOP and DOPM groups exhibited a significant increase in the positive area of IL‐10 compared to the other groups (Figure S14, Supporting Information), which confirms that DOPM implantation inhibited the relevant inflammatory signaling pathways.^[^ ^48^ ^]^

Overall, in vivo experiments demonstrated that the DOPM bone glue material has significant biocompatibility, excellent bone repair properties, and in vivo immune microenvironmental regulation abilities, which effectively promotes bone regeneration at bone defects in osteoporotic rats. This novel bone glue has potential applications in the treatment of osteoporotic bone defects in humans.

## 3 Conclusion

Inspired by the adhesive and robust fixation properties of concrete structures, we developed a novel biomimetic bone glue (DOPM) comprising a levodopa/oxidized chitosan hydrogel, which is the “cement,” and poly(lactic‐co‐glycolic acid) microspheres stabilized on an organic‐inorganic framework of nanohydroxyapatite, which is the “gravel.” DOPM exhibited excellent mechanical properties, biodegradability, and osteoconductive characteristics. In vitro and in vivo experiments confirmed that DOPM is biocompatible, induces osteogenic differentiation in mesenchymal stem cells, and modulates the immune microenvironment of aging macrophages, thus accelerating bone tissue repair and regeneration. This novel bone glue is highly promising for the field of bone tissue engineering and exhibits great potential for clinical applications.

## 4 Experimental Section

### Synthesis of DOPM

*Preparation of Ocs*: CS (low molecular weight; M_w_: 50 000–190 000 g mol^−1^; degree of deacetylation: 75%–85%) was purchased from Sigma–Aldrich (St. Louis, MO, USA). Briefly, a solution of CS (4 mmol) in 50 mL of HCl (pH 5.0) was continuously stirred for 24 h to achieve complete dissolution of the CS. Simultaneously, NaIO_4_ (Aladdin, Shanghai, China) was dissolved in deionized water in the dark, and the pH of the mixture was adjusted to 5.5. This mixture was added dropwise to the CS solution in the dark and continuously stirred for 24 h. The oxidation of CS involves the conversion of its abundant ‐OH groups to ‐CHO group. Hence, the solution was dialyzed using a polymer filtration membrane for 24 h, wherein the dialysis solvent was renewed every 8 h. The dialyzed product was lyophilized.

*Preparation of Levodopa/Ocs*: Levodopa (CAS No.: 59‐92‐7; C_9_H_11_NO_4_; M_w_: 197.19 Da) was purchased from Aladdin (Shanghai, China). N‐Hydroxysuccinimide ester (NHS; CAS No.: 6066‐82‐6; M_w_: 115.09 Da) and N‐ethyl‐N′‐(3‐dimethylaminopropyl)carbodiimide hydrochloride (EDC; CAS No.: 25952‐53‐8; M_w_: 191.70 Da) were obtained from MedChemExpress (New Jersey, USA). Ocs (400 mg) was dissolved in 80 mL of 0.5 M glacial acetic acid (Merck, Darmstadt, Hesse, Germany). The solution was stirred at room temperature for 40 minutes to ensure complete dissolution. NHS and EDC were added to a final concentration of 50 mM each. The pH was adjusted to 5.5 by adding HCl, and the reaction mixture was stirred at room temperature for 30 min, followed by the addition of 160 mg of Dopa and adjustment of the pH to 6.0. The solution was stirred at room temperature for an additional 4 h. The resulting product was dialyzed against deionized water using a dialysis membrane before being lyophilized.

*Preparation of mof@nHA*: Initially, 4.2516 g of ammonium dihydrogen phosphate (Aladdin, Shanghai, China; CAS No.: 7722‐76‐1; NH_4_H_2_PO_4_; *M_w_*: 115.03 Da) was dissolved in 35 mL of deionized water, and the pH was adjusted to 6 using 3 M HNO_3_. Subsequently, 4.4320 g of calcium chloride (Aladdin, Shanghai, China; CAS No.: 10043‐52‐4; CaCl_2_; *M_w_*: 110.984 Da) was added to the solution while stirring to ensure thorough mixing, and the pH was further adjusted to 5. After sufficient stirring, glutamic acid was introduced to the mixture and thoroughly mixed. The resulting solution was placed in a thermostatic air‐drying oven and reacted at 180 °C for 5 h, then cooled to room temperature. The mixture was ultrasonically dispersed to ensure homogeneity, followed by centrifugation and filtration. The supernatant was removed, and the precipitate was washed three times with deionized water to obtain a white pasty nHA solid. In the next step, 5.65 g of nHA was dissolved in 25 mL of water, and the pH was adjusted to 8. Subsequently, 0.3 g of gallic acid (Aladdin, Shanghai, China) was added, and the mixture was ultrasonically dispersed to ensure uniformity before being transferred to a three‐neck flask for reflux stirring. The reaction was carried out at 120 °C for 24 h, then cooled to room temperature, and the precipitate was collected by filtration. The collected precipitate was further washed three times with deionized water and absolute ethanol, followed by ultrasonic dispersion and high‐speed centrifugation to recover the final mof@nHA product.

*Preparation of PLGA/mof@nHA*: PLGA microspheres were synthesized from PLGA (CAS No.: 34346‐01‐5; C_5_H_8_O_5_; M_w_: 10000—20 000 Da;, MedChemExpress, New Jersey, USA) using the microfluidic technique. Customized coaxial needles (25 G, 18 G) with internal metal channels (0.26 mm i.d. × 0.51 mm o.d.) and external metal channels (0.84 mm i.d. × 1.27 mm o.d.) connected via polyvinyl chloride (PVC) pipe connectors were used. First, 0.75 g gelatin (Aladdin, Shanghai, China) was dissolved in 10 mL deionized water. Concurrently, 0.2 g PLGA was dissolved in 10 mL dichloromethane (Aladdin, Shanghai, China) to prepare the PLGA solution. Using a syringe, 2 g of the gelatin solution was slowly injected into 6 g of the PLGA solution. After ultrasonic agitation for 5 min, 0.05 g mof@nHA was added and evenly mixed. A polyvinyl alcohol (PVA, Aladdin, Shanghai, China) solution served as the continuous phase flowing through an external channel, with a fixed two‐phase flow rate ratio of 1:18. The resulting microspheres were then added to 1000 mL of a 0.75% PVA solution and gently stirred overnight to remove the gelatin, yielding the PLGA/mof@nHA mixed solution. The supernatant was discarded, and the microspheres were repeatedly washed and resuspended three times with PBS. The product was then freeze‐dried at a low temperature for 48 h and stored in a sealed container at 4 °C. Finally, the collected PLGA/mof@nHA microspheres were lyophilized at −80 °C for 1 d.

*Optimization of the Glue‐to‐Spheres Ratio*: To determine the bone glue‐to‐PLGA/mof@nHA ratio yielding the maximum adhesion strength, five samples were prepared with the following flue‐to‐spheres ratios: (1) 1 g DO, (2) 1 g DO:0.75 g PM; (3) 1 g DO:0.50 g PM; (4) 1 g DO:0.25 g PM; and (5) 1 g DO:0.10 g PM.

### Characterization of DOPM

*Fourier‐Transform Infrared Spectroscopy*: The structural changes in the samples were evaluated with a Fourier‐transform infrared spectrometer (PerkinElmer Spectrum 3 FT‐IR, Waltham, Massachusetts, USA).

*SEM and AFM*: A field emission gun was used as the electron source. Each sample was placed on a slide, which was mounted on an aluminum rod using double‐sided carbon tape. The completely dried samples were sputter‐coated with Pt and were observed under an SEM (Hitachi S‐4800, Tokyo, Japan) at an accelerating voltage of 12 kV. The topological morphology of the material surface was observed using AFM (Dimension ICON, Bruker, Billerica, Massachusetts, USA).

*Rheological Studies and XPS Analysis*: The rheological behavior of DOPM, i.e., the changes in G′ and G″, was characterized using a rheometer (Anton Paar MCR 302, Graz, Austria) at 37 °C with a gap of 0.5 mm., XPS analysis of the samples was performed using a 250 Xi spectrometer (Thermo Scientific ESCALAB Xi+, Waltham, Massachusetts, USA).

*Mechanical Performance*: Shear, tensile, and stress‐strain, bending, and torsion tests of the samples were performed using a mechanical testing machine (Hengyi, Shanghai, China).

### In Vitro Experiments

*Extraction of Rat BMSCs*: Rat BMSCs were isolated and cultured from the bone marrow of 21‐month‐old male SD rats using a method approved by the Ethics Committee of the First Affiliated Hospital of the University of Science and Technology of China,Hefei. Briefly, rat femurs were isolated under sterile conditions. First, both ends of the femur were gently cut off using ophthalmic scissors. The bone marrow cavity of the femur was then repeatedly rinsed with phosphate‐buffered saline (PBS) with a 5‐mL sterile syringe until it exhibited a whitish color. The bone marrow fluid was collected, filtered using a 40‐µm cell sieve, and centrifuged at 1500 rpm for 10 min. The cell pellet was incubated in Dulbecco's modified Eagle medium (DMEM, Gibico, Waltham, Massachusetts, USA), comprising 10% fetal bovine serum (FBS), 100 U mL^−1^ penicillin G, and 0.1 mg mL^−1^ streptomycin, at 37 °C, 5% CO_2_, and saturated humidity. The cells were cultured to the third generation, and their expression of CD44, CD73, CD45, CD11b, and CD105 antibodies (1:100; Abcam, Cambridge, England) was evaluated using flow cytometry. The positive expression of CD44, CD73, and CD105 (>90%) and the negative expression of CD45 and CD11b (< 5%) confirmed the identity of BMSCs, which were then used for the subsequent in vitro experiments.

*Cell Cultures*: A BB 150 CO_2_ incubator (BB150‐2TCS‐L, Thermo Scientific, Waltham, Massachusetts, USA) at 5% CO_2_ and saturated humidity equipped with a thermostat at 37 °C was used. All equipment was sterilized with ethanol under UV light overnight prior to using it for growing cell cultures in sterile PBS. The RAW264.7 cells (Procell, Wuhan, China) were cultured using DMEM (HyClone, Logan, Utah, USA), whereas the BMSCs were cultured using Minimum Essential Medium (α‐MEM), wherein 10% FBS (Gibco) and 1% penicillin/streptomycin (Beyotime, Shanghai, China) were added to both cultures. The culture medium was renewed every 1–2 d. Cell passaging was performed when the cells grew completely adherent to the wall and reached 80%–90% fusion. The cell culture fluid was removed via negative pressure suction, and the cell pellet was washed twice with PBS. Trypsin (1 mL) was added to the pellet, followed by shaking and digestion for 1 min at 37 °C. Next, 2 mL of high‐glucose DMEM was added to terminate the digestion. The cells were uniformly suspended in the medium by blowing the adherent cells with a pipette gun and were cultured in a 1:2 ratio for cell passaging.

*Biocompatibility Assay*: The cytobiocompatibility of DOPM was assessed by the CCK‐8 method (Solarbio, Beijing, China). The RAW264.7 cells and BMSCs in the logarithmic growth phase were suspended in culture medium and the sample solution was observed under the microscope. The two types of cells were then seeded in 96‐well plates at a density of 4 × 10^3^ cells well^−1^, and incubated. The cells that adhered to the wall were collected and incubated for 1, 3, and 7 d. Next, 100 µL of the CCK‐8 solution was added to the 96‐well plate and incubated at 37 °C for 2 h. The 96‐well plate was then placed into an enzyme labeling instrument (BIO‐TEK, Winooski, Vermont, USA) and the optical density (OD) was measured at 450 nm to evaluate the cell activity.

Subsequently, the RAW264.7 cells and BMSCs were seeded in 24‐well plates at a density of 2×10^4^ cells well^−1^ and incubated with the extraction solution for 3 d. Then, the live and dead cells were simultaneously evaluated using a fluorescent live/dead cell double staining kit (Beyotime, Shanghai, China). The staining solution was prepared by dissolving 5 µL of calcein (AM) and 15 µL of propidium iodide (PI) in 5 mL PBS. This solution was added to the cells, which were then incubated for 30 min. The fluorescence staining images of the live/dead cells were recorded using an inverted fluorescence microscope at 490 and 535 nm.

*Preparation of the Conditioned Media*: To investigate whether DOPM can affect BMSC differentiation by regulating macrophage polarization, the supernatant from RAW264.7 cells cultured on various substrates was collected, centrifuged, and filtered to remove any cell debris and impurities. Specifically, the RAW264.7 cells in the logarithmic growth state were inoculated at 4 × 10^5^ cells/well in the upper chamber of a Transwell device (Corning, New York, USA), whereas the samples were placed in the lower chamber. After complete attachment of the cells to the wall, the culture medium was treated with LPS (1 µg mL^−1^) for 12 h. The supernatant was collected and filtered under aseptic conditions to remove cellular debris and impurities. These supernatants were subsequently mixed with fresh low‐glucose DMEM in equal proportions to prepare a macrophage conditioned medium (MCM). Finally, osteogenic components (10 mM β‐glycerophosphate, 0.1 µM dexamethasone, and 0.25 mM ascorbate) were added to MCM to prepare an osteogenic‐conditioned medium.

In vitro *osteogenesis*: All equipment was sterilized via Co‐60 irradiation. BMSCs were seeded in 6‐well plates (NEST, China) at a density of 1 × 10^4^ cells per well, When the cell density reached 60%, the cell culture medium was replaced with the conditioned media. The medium was renewed every 3 d. After 7 d of culture, the cells were washed 1–2 times with PBS and fixed with 4% paraformaldehyde. The samples were then stained using an alkaline phosphatase (ALP) staining kit (Beyotime, Shanghai, China) according to the manufacturer's instructions. The cell morphology was observed under a light microscope. The ALP activity was investigated using the following procedure: after 7 d of culture, the medium was discarded, and the cells were incubated with 0.1% TritonX‐100 for 1 min and centrifuged. Their ALP activity was detected using an AKP/ALP kit (Beyotime, Shanghai, China). After 21 d of culture, BMSCs were fixed with 4% paraformaldehyde and stained with an alizarin red staining kit (Cyagen, China) according to the manufacturer's instructions. The cells were then observed under a microscope. To determine the amount of calcium deposits, 1 mL of 10% cetylpyridinium chloride was added to each well after staining to dissolve the stained calcium nodules. After 1 h, the absorbance was measured at 562 nm using an enzyme marker.

*Immunofluorescence*: The cells were intervened according to the experimental requirements, washed 2–3 times with PBS, and fixed with 4% paraformaldehyde for 10 min. The paraformaldehyde was discarded, and the cells were washed with PBS, permeabilized with 0.1% Triton X‐100 (Beyotime, Shanghai, China), and treated with an immunostaining blocking solution (Beyotime, Shanghai, China). The cells were incubated overnight with primary antibodies at 4 °C and then with fluorescent‐labeled secondary antibodies at 37 °C for 1 h in the dark. The cell nuclei were stained using a ghost pen cyclic peptide (Yeasen, Shanghai, China) and sealed with an anti‐quenching sealer. The fluorescent‐labeled cells were observed under a laser confocal microscope (Zeiss, Oberkochen, Baden‐Württemberg, Germany) in the dark, and the fluorescence intensities of the target proteins were assessed using the Image J program (Bethesda, Bethesda, Maryland, USA).

*Flow Cytometry*: The treated cells were washed with PBS, digested with 0.25% trypsin digest (Servicebio, Wuhan, China), and centrifuged. BSA was added to the cell pellet (Merck, Darmstadt, Hesse, Germany) to afford a cell suspension. A fluorescein‐labeled flow‐through antibody was added to the cell suspension, followed by incubation in the dark at 4 °C for 30 min. The cell suspension was centrifuged, the supernatant was discarded, and the cells were washed and resuspended in PBS. The sample obtained was analyzed using a BD FACSCelest flow cytometer (Franklin Lakes, New Jersey, USA) and the Flowjo‐v10.8.1 software (Ashland, Oregon, USA).

*RT‐PCR Assay*: The cells were cultured according to the procedure described in Section 4.3.2., and the expression of target mRNA in cells was detected by qRT‐PCR using the following procedure: The total RNA of the cells was extracted using the Simple P Total RNA Extraction Kit (EZBioscience, California, USA) according to the manufacturer's instructions. The RNA concentration was determined using the Nanodrop 2000 spectrophotometer (Thermo Scientific, Massachusetts, USA). cDNA was synthesized by reverse transcription using the HiScript III‐RT SuperMix for qPCR kit (Nanjing, China). qRT‐PCR was performed using the ChamQ Universal SYBR qPCR Master Mix kit (Nanjing, China) according to the manufacturer's instructions. The relative gene expression of the cells in comparison to the expression of GAPDH was calculated using the 2^−ΔΔCt^ method. The primer sequences were shown in Table S3 (Supporting Information).

### Western Blot Assay

The RAW264.7 cells and BMSCs were plated separately according to the procedure described in Section 4.3.5. The plates were placed on ice and washed with pre‐cooled PBS (×3). The total protein was extracted from the cells by adding a RIPA lysate comprising protease inhibitors. The protein concentration was determined using the BCA Protein Concentration Assay Kit (California, USA) according to the manufacturer's instructions. The concentration of each sample protein was adjusted with a 5× protein sampling buffer, boiled in a water bath for 5 min, and stored in a refrigerator at −20 °C. The proteins were then separated via electrophoresis using an SDS‐PAGE gel (FUDE BIOLOGICAL, Nanjing, China) and wet‐transferred to a PVDF membrane. TBST and 5% skimmed milk were added to the membrane, which was then incubated at room temperature for 1 h in a shaker. The membrane was washed with TBST (×3). Primary antibodies were diluted according to the manufacturer's instructions and incubated overnight with the PVDF membrane at 4 °C upon continuous shaking. The primary antibodies were recovered and TBST (×3) was added to the membrane, which was shaken for 5 min each time. Secondary antibodies were diluted in the ratio of 1:5000 and incubated with the PVDF membrane at 37 °C for 2 h. The membrane was then washed (×3), and the excess water was removed with absorbent paper. Next, the membrane was placed in a light‐blocking cassette containing an enhanced chemiluminescent solution, reacted for 2 min, and analyzed using a chemiluminescent gel imaging system (Proteinsimple, California, USA). The gray values of the protein bands were analyzed using AlphaEaseFC software (California, USA).

### Transcriptomic Analysis

The macrophages were incubated with LPS (1 µg mL^−1^) and the DOPM bone glue for 24 h. The total cellular RNA was extracted using Trizol (Thermo Scientific) followed by freezing at −80 °C. The RNA purity was determined using the NanoDrop 2000 spectrophotometer, and the RNA integrity was assessed using an Agilent 2100 bioanalyzer (Agilent Technologies, Santa Clara, CA, USA). The libraries were sequenced using the Illumina Novaseq 6000 sequencing platform to afford 150 base‐pair double‐ended reads. Hence, clean reads were obtained using fastp software (Beijing, China) for subsequent data analysis. The fragments per kilobase of transcript per million mapped reads (FPKM) were calculated using HISAT2 software (California, USA), and sample biological replicates were evaluated by HTSeq‐count (Heidelberg, Germany) and R (v3.2.0). Differentially expressed gene (DEG) analysis was performed using DESeq2 software (v1.34.0, Bioconductor), where DEGs exhibit q‐value < 0.05 and foldchange > 2 or < 0.5. DEG analysis of the different samples was also performed using R (v3.2.0). Subsequently, GO, KEGG, Pathway, Reactome, and WikiPathways enrichment analysis of DEGs was conducted with the hypergeometric distribution algorithm, which was used for screening significantly enriched functional entries. R (v3.2.0) was used to plot bar charts, chord charts, or circular maps for significantly enriched functional entries. Gene set enrichment analysis was performed using GSEA software (v5.0, Bioconductor). The genes were sorted into predefined sets according to the degree of differential expression. Next, it was evaluated whether the predefined set of genes was enriched at the top or bottom of this sorted table.

### In Vivo Experiments

*Experimental Design*: All procedures, including surgery and preoperative/postoperative care, were performed according to protocols approved by the Ethics Committee of the First Affiliated Hospital of Science and Technology of China.

*Ex Vivo Measurements of the Adhesion of Bone Glue to Porcine Bone*: The adhesion strength of the DOPM bone glue was evaluated on isolated fresh pig femurs procured from local butchers. The surface soft tissue of the femurs was stripped off, and the femoral heads were sawed off. Then, the broken bone ends were injected with 100 µL of the DOPM bone glue and coated uniformly. The fracture breaks were spliced, and the adhesive was allowed to fully solidify at 37 °C. The femurs were then pretreated overnight in PBS at 37 °C, perforated, and threaded to a 10‐kg weight at the end of the femoral head.

*Subcutaneous Implantation Model*: Male Sprague‐Dawley (SD) rats aged twenty‐one months were used to model the dorsal subcutaneous implantation of stents. All equipment was sterilized with ethylene oxide, and all experimental procedures were completed under aseptic conditions. The SD rats were anesthetized with 10% chloral hydrate (0.3 mL/100 g body weight) via an intraperitoneal injection, and the hair on their backs was removed with a razor and a depilatory agent. The surgical area was disinfected with iodophor, and a longitudinal incision of ≈2 cm was made in the middle of the back of each rat. The skin was gently lifted up from both sides of the incision with toothed tweezers, and the skin and subcutaneous tissue were bluntly separated from the incision using vascular forceps to form small subcutaneous pouches on both sides of the rat's back. The bone glue (100 µL) was placed into both the subcutaneous pouches, and the incision was closed with a 4‐0 absorbable suture. For 3 d after the surgery, the rats were intramuscularly injected with penicillin and kept warm. All experimental animals were in good condition after the surgery, and the dorsal incisions healed well with no obvious signs of infection. The rats were euthanized at 1, 2, and 4 weeks after the surgery, and the subcutaneous area of the implanted adhesive was dissected and exposed for gross observation. At a certain timepoint, the dry weight (W_1_) and the initial dry weight (W_0_) were measured, and the remaining weight percentage was calculated using the following equation:

(1) $$\mathrm{Remaining}\;\mathrm{weight}\;\mathrm{percentage}=\left(W_{0}-W_{1}\right)\;\;/W_{0}\times 100\%$$

Local specimens were also removed from the SD rats and were fixated with a neutral universal tissue fixative, embedded with paraffin, sectioned, and stained with HE for subsequent pathological evaluation.

### Animal Models

An in vivo bone defect repair experiment was conducted on aged SD rats (male, 21 months old, average weight of 550 ± 150 g). The rats were randomly divided into six groups, each consisting of six individuals. Additionally, to establish the presence of osteoporosis in aged SD rats, a control group comprising SD rats (male, 2 months old, average weight of 250 ± 50 g) was included. All animals were purchased from the Animal Experiment Center of the First Hospital Affiliated to the University of Science and Technology of China. All experimental procedures were approved by the Ethics Committee of the First Affiliated Hospital of the University of Science and Technology of China (2024‐N (A) −121). The osteoporosis model was confirmed by micro‐CT. A bone defect surgery was performed on the rats. Layered incisions were made into the rat skin to expose the lateral femoral condyle, and a defect of 5‐mm diameter and 5‐mm depth was made using an electric drill. Next, the bone glue was added to the bone defect. The rats were euthanized in batches after 2, 4, and 8 weeks, and the femoral specimens were removed intact and fixed with 4% paraformaldehyde until further analysis.

### Continuous Osteogenic Fluorescent Labeling

A two‐color sequential fluorescent labeling method was used to mark the process of new bone formation and mineralization. Alizarin red S (30 mg kg^−1^, Beyotime, Shanghai, China) and Calcein (30 mg kg^−1^, Beyotime, Shanghai, China) were intramuscularly injected into the rats at 6 and 8 weeks after the operation, respectively. The animals were euthanized, and samples were collected one week after the final injection of Calcein (on the 9th week) to ensure the dye was well incorporated into the bone tissue.

### Micro‐CT Analyses

The SD rats were examined by micro‐CT with the following parameters: operating voltage, 80 KV; current, 385 µA; exposure time, 240 ms; scanning thickness, 18 µm. 3D Creator software was used to reconstruct the image in three dimensions using the Feldkamp algorithm. The regions of interest (ROI) within the femoral condylar defect were manually selected. The bone volume/tissue volume (BV/TV), trabecular width (Tb.N), trabecular number (Tb.Th), and bone mineral density (BMD) of ROIs were evaluated using CTVox software.

### Histological and Immunohistochemical Analyses

Sections of the SD rat dorsal subcutaneous implant model specimens were evaluated using HE staining to identify any inflammatory foreign body reactions caused by the bone glue. Panoramic images of whole sections were captured using a digital section scanner (KF‐PRO‐120, KFBIO, Zhejiang, China). After CT scanning, the femoral specimens were decalcified with 10% ethylenediaminetetraacetic acid (Sigma–Aldrich) for 1 month, embedded with paraffin, and sectioned using a sectioning saw (SP1600, Leica, Wetzlar, Germany). All sections were evaluated by HE, Masson, and immunohistochemical staining. The primary antibody and the corresponding HRP‐labeled secondary antibody were incubated for immunohistochemical staining.

### Statistical Analysis

All quantitative data were expressed as mean ± standard deviation and were analyzed using the Kolmogorov Smirnov method for normality, one‐way analysis of variance (ANOVA), and t‐test with GraphPad Prism 9.2 software (California, USA). Tukey's test was used for post‐hoc analysis to compare multiple treatments. Moreover, p < 0.05 was considered statistically significant, and *: p < 0.05; **: p < 0.01; ***: p < 0.001.

## Conflict of Interest

The authors declare no conflict of interest.

## Supporting information



Supporting Information

## Data Availability

The data that support the findings of this study are available from the corresponding author upon reasonable request.
