# Supplementary material for: Concrete‐Inspired Bionic Bone Glue Repairs Osteoporotic Bone Defects by Gluing and Remodeling Aging Macrophages
Source: Adv Sci (Weinh). 2024 Oct 25;11(48):2408044. doi: 10.1002/advs.202408044 (PMC11672322; doi:10.1002/advs.202408044)
Supplement: Supplementary file 1 — Supporting Information [file ADVS-11-2408044-s001.docx]

Supporting Information for

Concrete-Inspired Bionic Bone Glue Repairs Osteoporotic Bone Defects by Gluing and Remodeling Aging Macrophages

*Chong Li^#^, Wei Xu^#^, Lei Li^#^, Yonghui Zhou, Gang Yao, Guang Chen, Lei Xu, Ning Yang, Zhanjun Yan, Chen Zhu, Shiyuan Fang*, Yusen Qiao*, Jiaxiang Bai*, Meng Li**

Dr. C. Li, Dr. W. Xu, Dr. L. Li, Dr. G. Yao, Dr. G. Chen, Dr. L. Xu, Dr. N. Yang, Dr. C. Zhu, Prof. S. Fang, Prof. Dr. J. Bai, Prof. M. Li

Department of Orthopedics, Centre for Leading Medicine and Advanced Technologies of IHM, The First Affiliated Hospital of USTC, Division of Life Sciences and Medicine, University of Science and Technology of China, Hefei, 230022, Anhui, China

**E-mail:** **jxbai1995@ustc.edu.cn (J. Bai)**; limengustc@163.com (M. Li).

Prof. Y. Qiao

Department of Orthopedics, The First Affiliated Hospital of Soochow University, 188 Shizi Road, Suzhou 215006, Jiangsu, China

1. **mail:** qiaoyusen8612@suda.edu.cn (Y. Qiao)

Dr. C. Li, Prof. S. Fang

Department of Orthopedics, Anhui Provincial Hospital Affiliated to Anhui Medical University, Hefei, 230022, Anhui, China

**E-mail:** fangsyustc@163.com (S. Fang)

Dr. Z. Yan

Department of Orthopedics, The Ninth People's Hospital of Suzhou; Suzhou 215006, Jiangsu,

China

Figures and Legends

Fig. S1.

**Control**

**Dopa**

**Ocs**

**DO**

**DOP**

**DOPM**


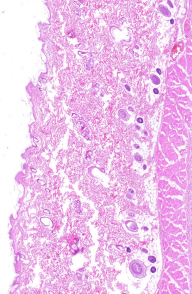

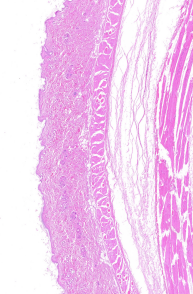

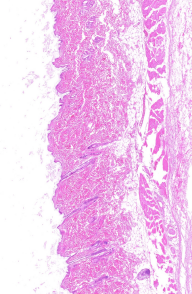

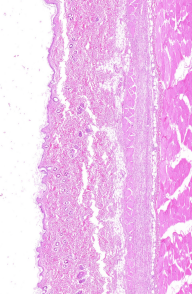

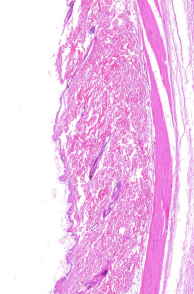

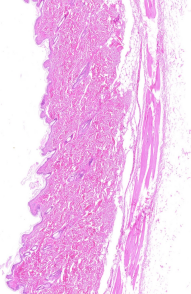


**Dopa**

**Ocs**

**DO**

**DOP**

**DOPM**


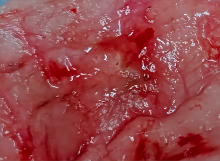

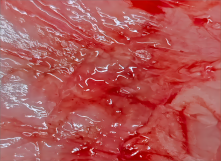

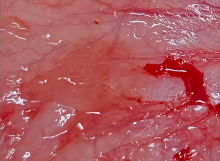

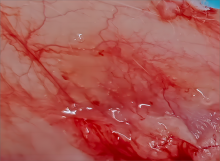

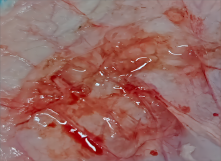

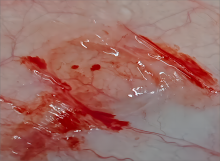

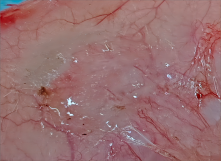

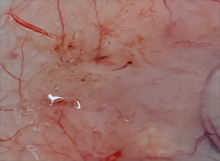

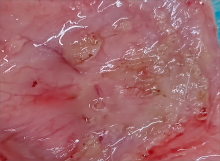

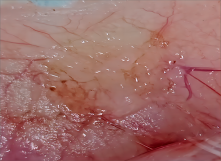

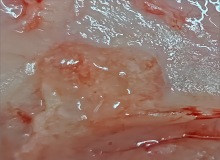

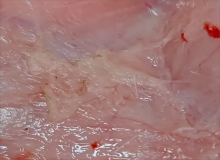

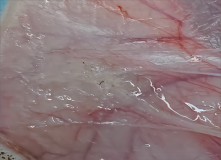

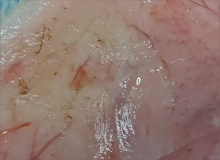

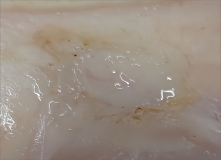


**1w**

**2w**

**4w**


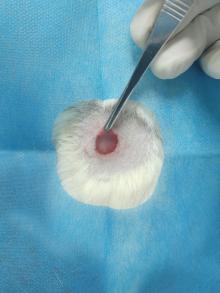

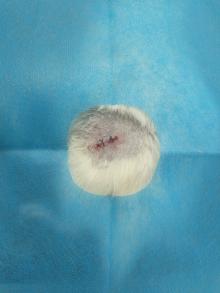


**a**

**b**

**c**

**Fig. S1.** (a) Dorsal subcutaneous implantation experiment in SD rats. (b) H&E staining of the implant site 4 weeks after implantation. (c) H&E staining of the implanted samples at the subcutaneous site at 1, 2, and 4 weeks(scale bar=200 µm).

Fig. S2.


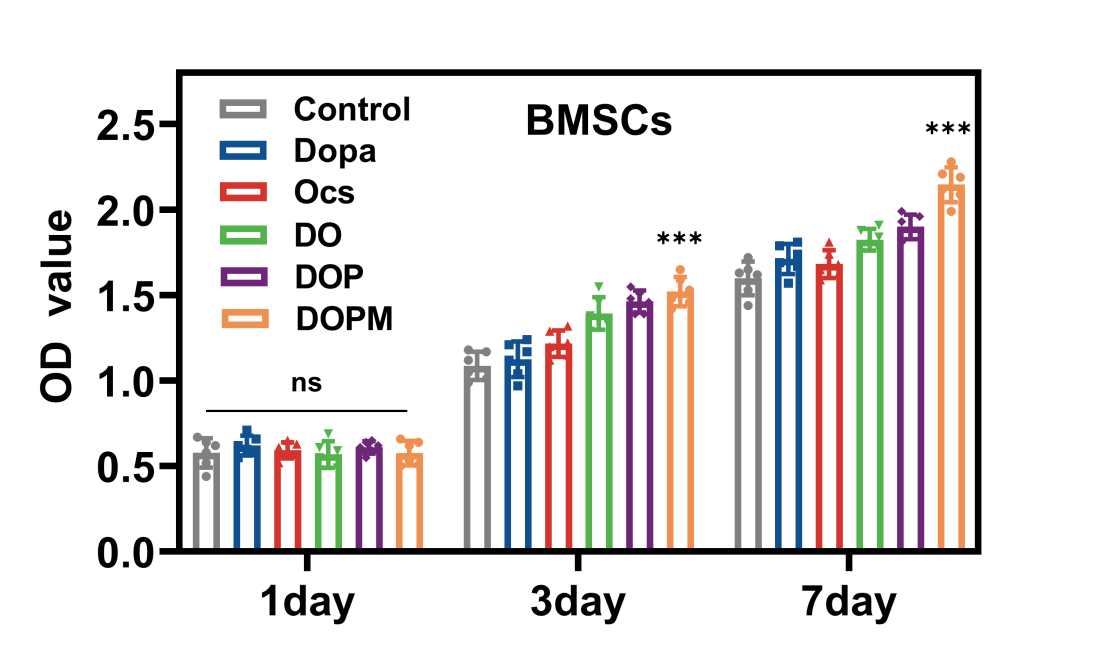


**Fig. S2.** Survival of BMSCs was assessed by CCK-8 after 1, 3 and 7 days of incubation with different sample extracts (n=6, data shown represent mean ± SD. *p < 0.05, **p < 0.01, ***p < 0.001, ns, no significance).

Fig. S3.


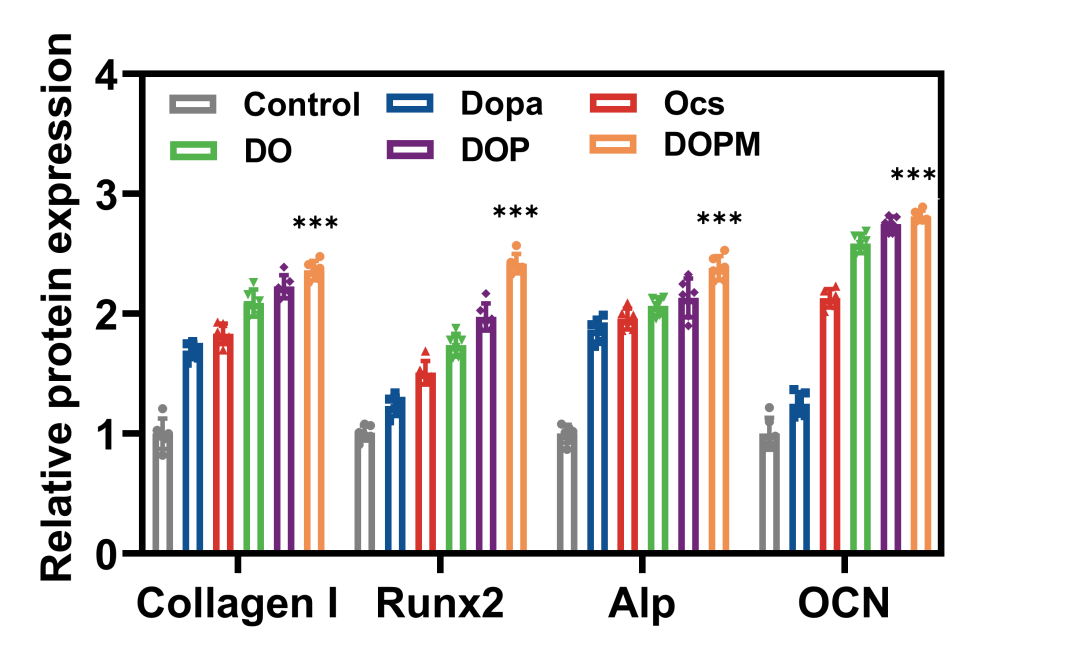


**Fig. S3.** Western blotting of Collagen I, RUNX2, ALP and OCN proteins (n=6, data shown represent mean ± SD. *p < 0.05, **p < 0.01, ***p < 0.001, ns, no significance).

Fig. S4.


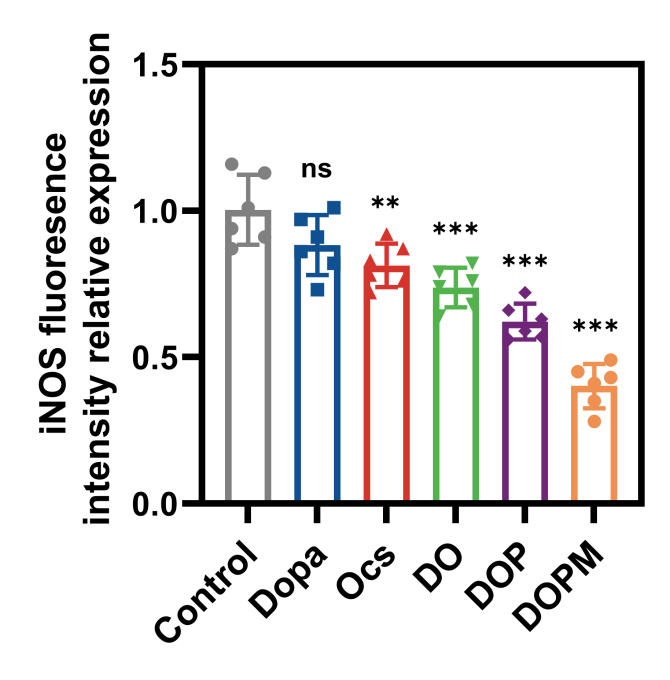

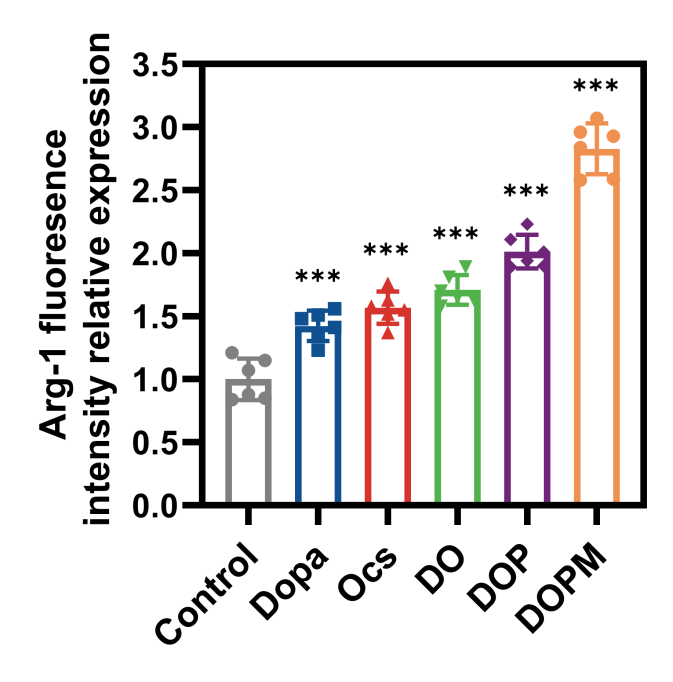


**Fig. S4.** Quantitative analysis of immunofluorescence intensity of iNOS and Arg-1 (n=6, data shown represent mean ± SD. *p < 0.05, **p < 0.01, ***p < 0.001, ns, no significance).

Fig. S5.


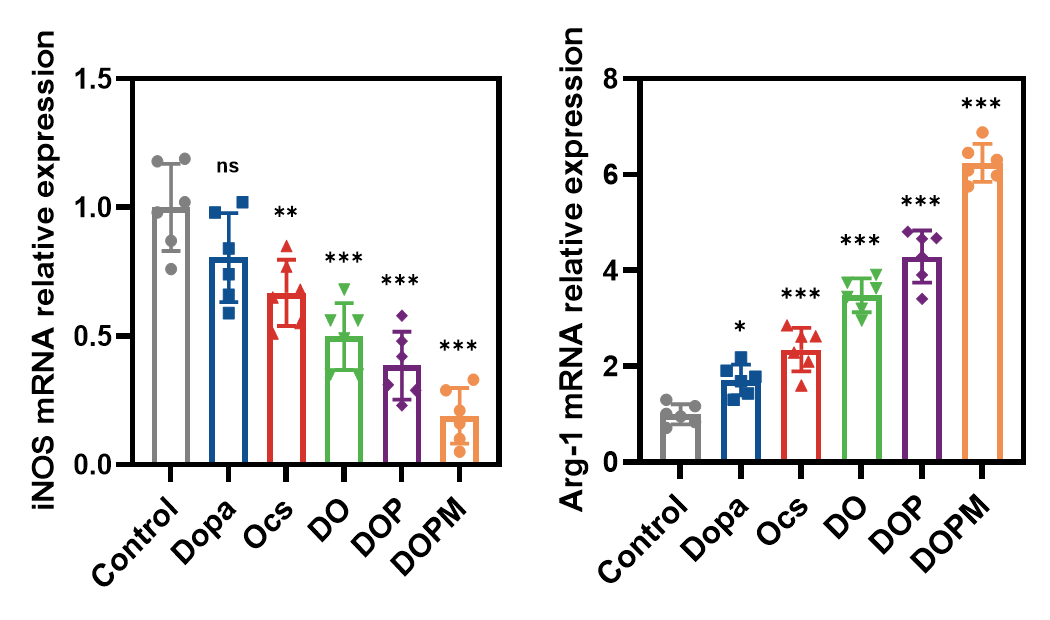


Fig. S5. The mRNA expression levels of iNOS and Arg-1 were analyzed through RT-PCR experiments (n=6, data shown represent mean ± SD. *p < 0.05, **p < 0.01, ***p < 0.001, ns, no significance).

Fig. S6.


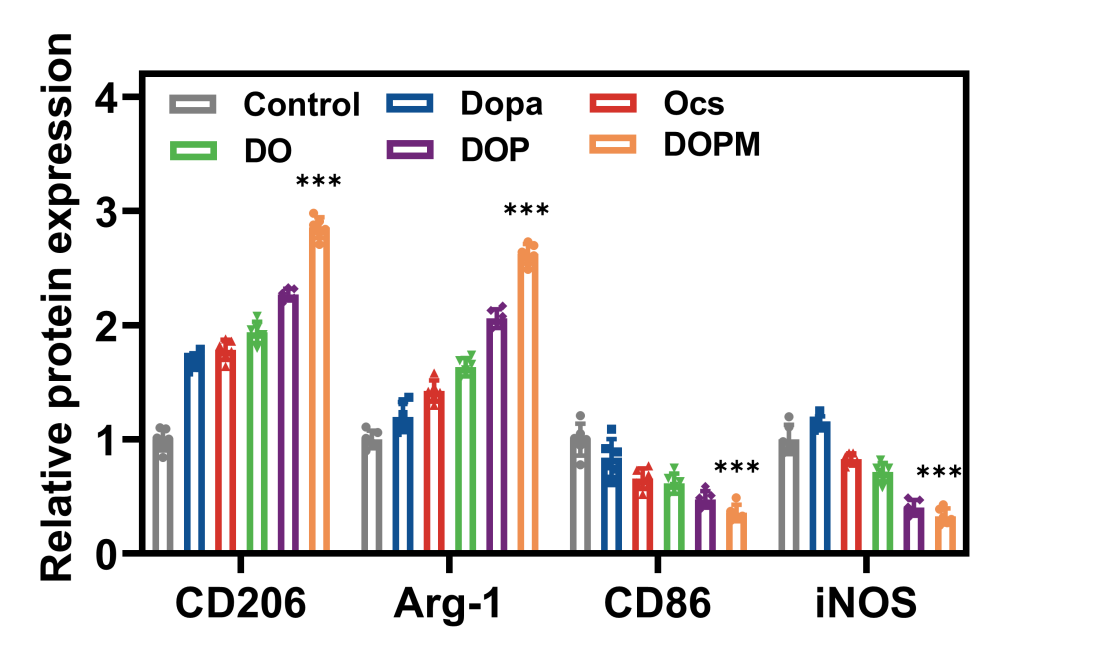


**Fig. S6.** Western blotting of CD206, Arg-1, CD86 and iNOS proteins (n=6, data shown represent mean ± SD. *p < 0.05, **p < 0.01, ***p < 0.001, ns, no significance).

Fig. S7.


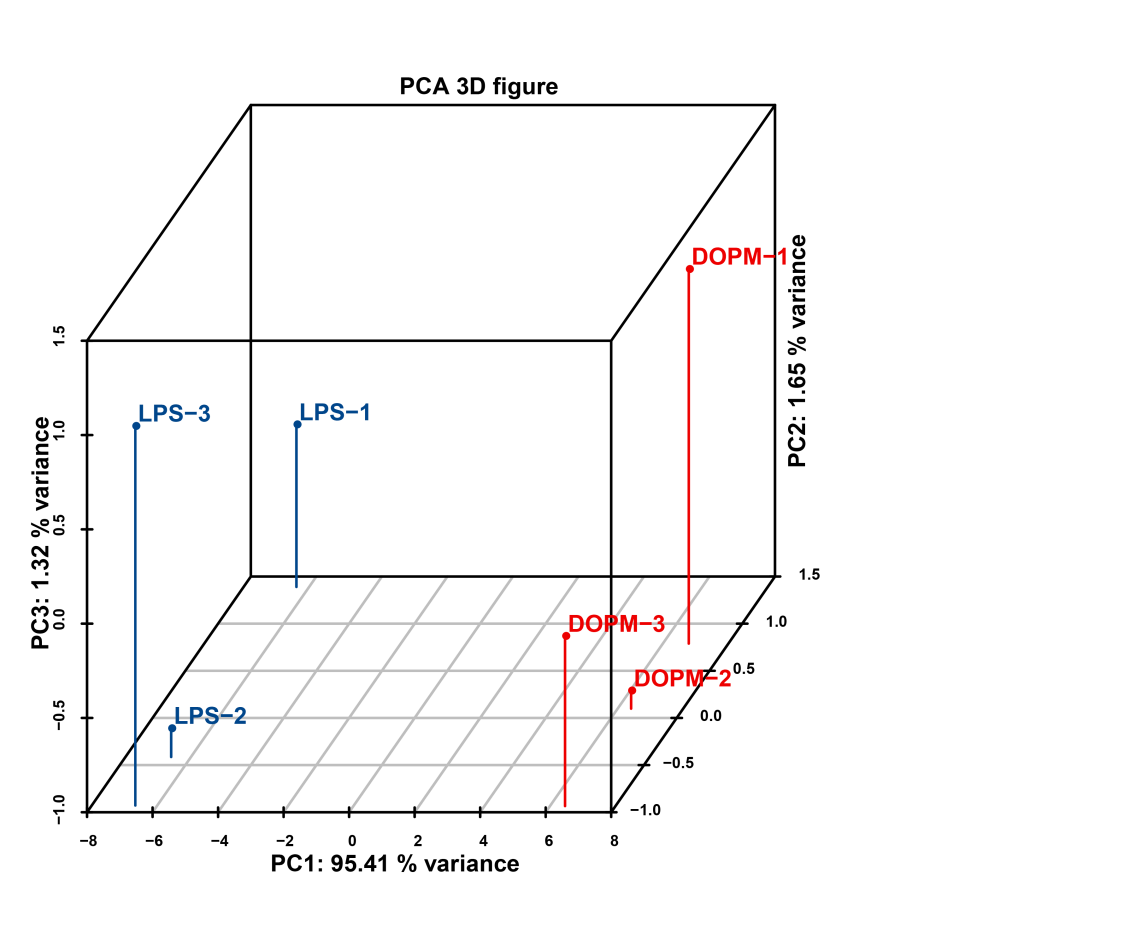


**Fig. S7.** PCA analysis of DEGs in LPS and DOPM samples.

Fig. S8.


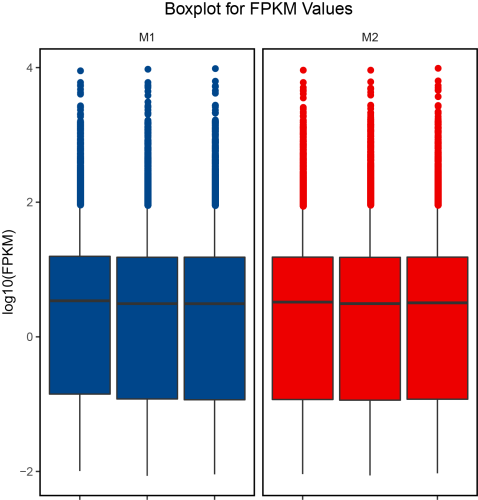

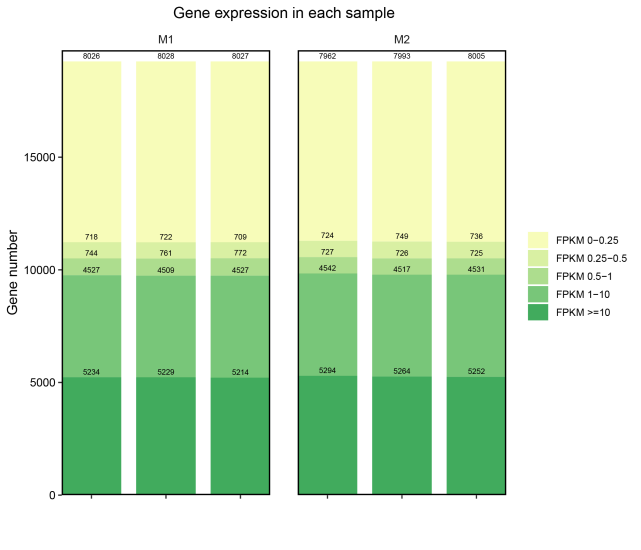


**a**

**b**

**Fig. S8.** Distribution of gene expression in the samples. (a) FPKM box plot of gene expression in samples. (b) Stacked histogram of the distribution of gene expression in the samples.

Fig. S9.


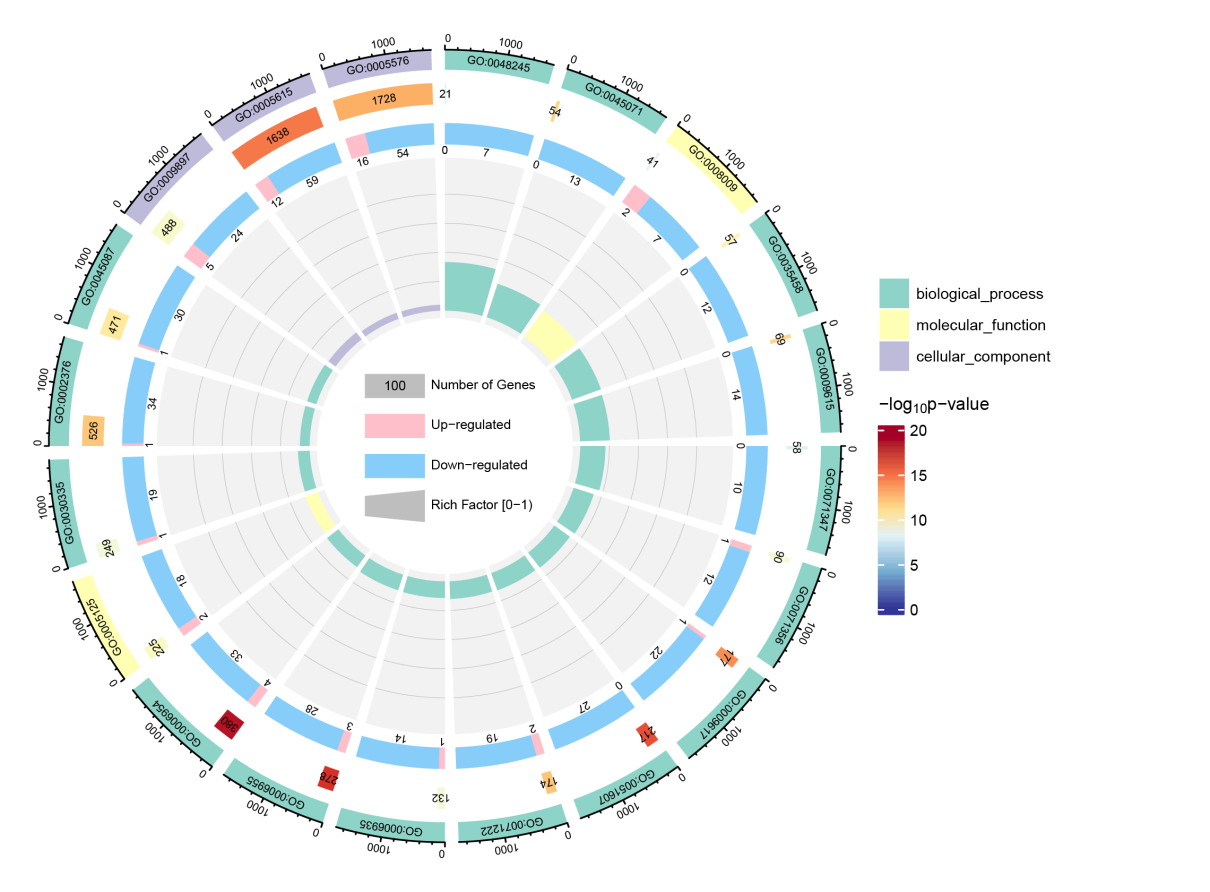


**Fig. S9.** GO enrichment analysis circle of differentially expressed genes in DOPM and LPS groups.

Fig. S10.

Fig. S10. Quantification of Tb.N, Tb.Th and Tb.Sp in aged SD rats (n=6, data shown represent mean ± SD. *p < 0.05, **p < 0.01, ***p < 0.001, ns, no significance).

Fig. S11.

**Defect**

**Exposure**

**Suture**


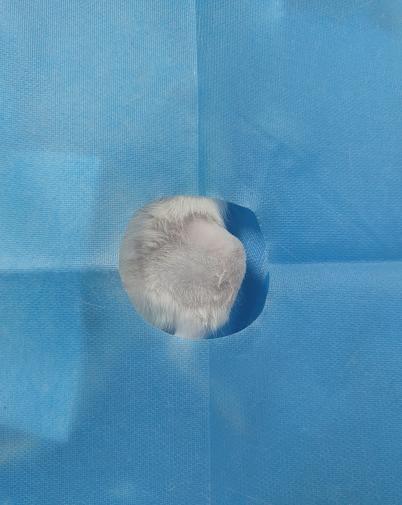

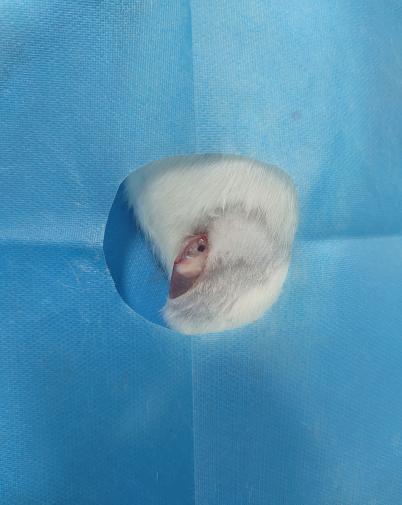

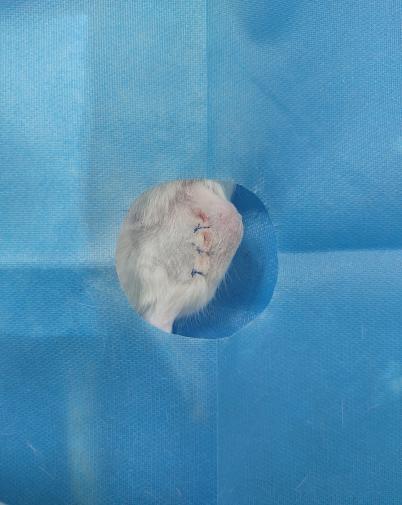


Fig. S11. Modeling of femoral condylar bone defects in aged osteoporosis model.

Fig. S12.


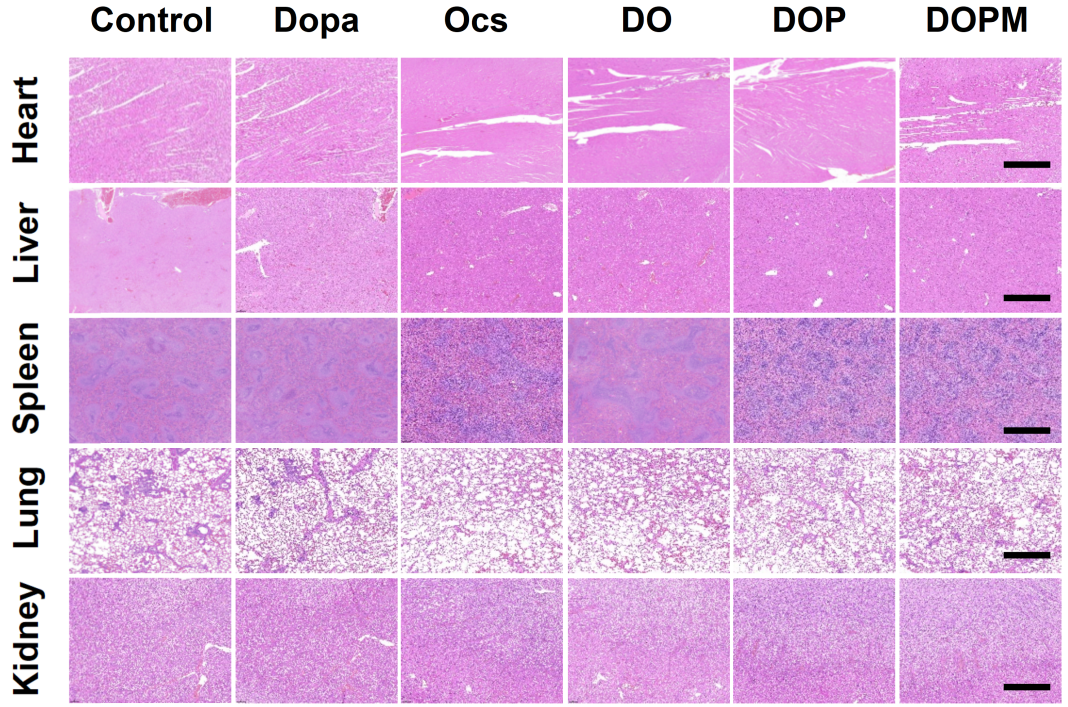


Fig. S12. H&E staining of different groups of major viscera at 8 weeks of implantation: heart, liver, spleen, lungs and kidneys. (scale bar=200 µm).

Fig. S13.


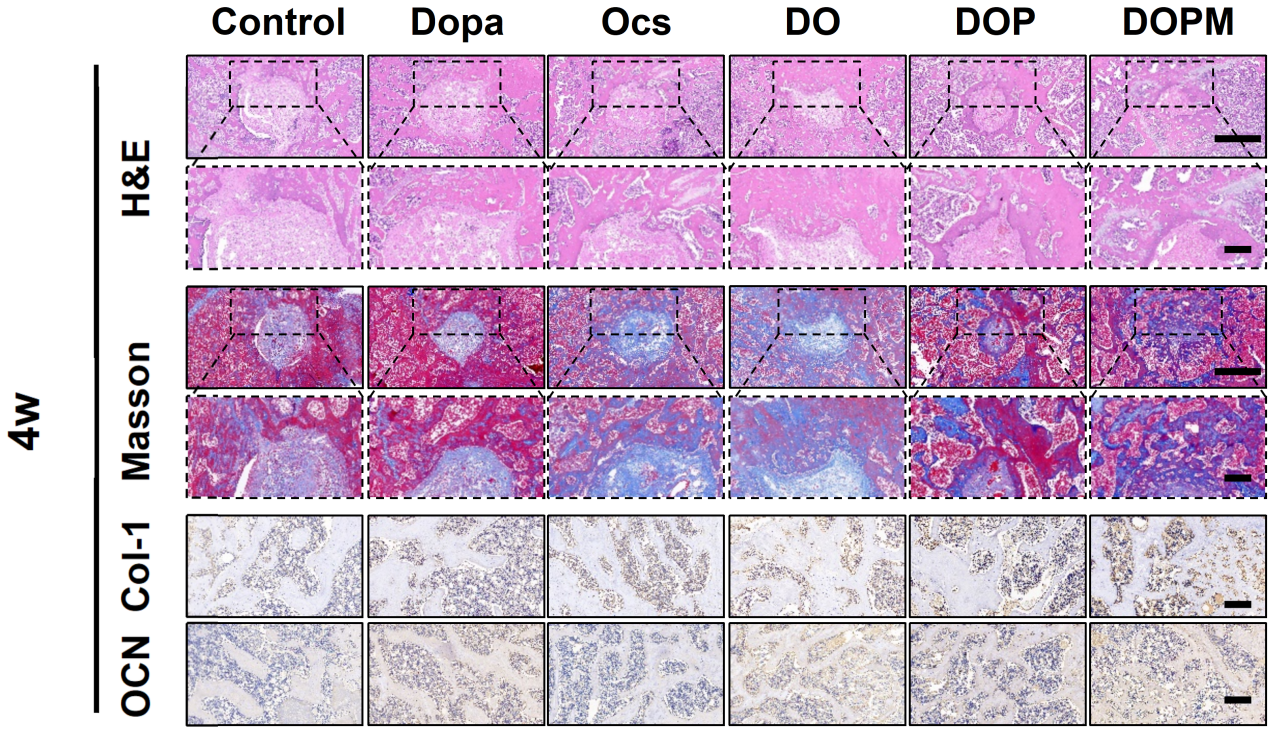


Fig. S13. Representative images of bone tissue with H&E and Masson staining after 4 weeks of treatment; scale bar = 100 μm (top) and 20 μm (bottom). Col-1 and OCN immunohistochemical staining of bone tissue after 4 weeks. scale bar = 20 μm.

Fig. S14.


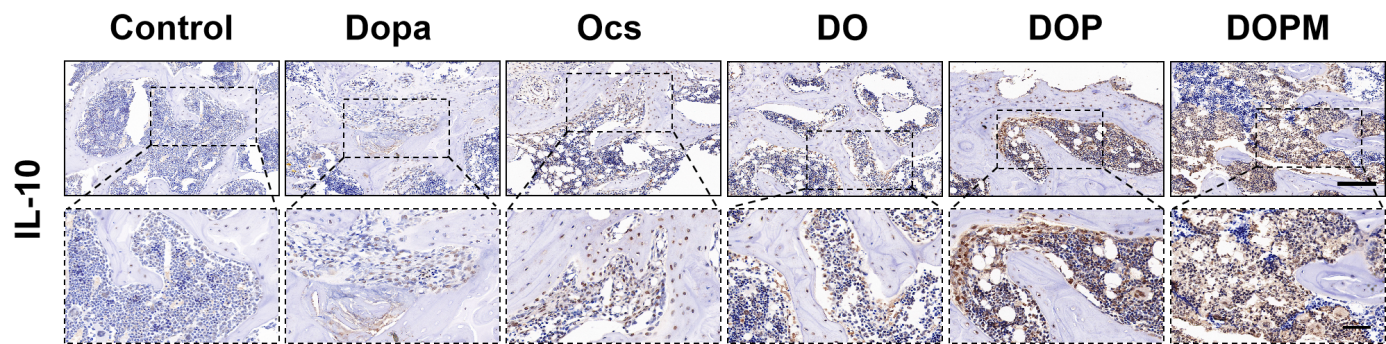


Fig. S14. Immunohistochemical staining of surrounding tissues was performed to evaluate the expression level of IL-10; scale bar = 100 μm (top) and 20 μm (bottom).

Table S1.

Material grouping and abbreviation

Group

Function

Abbreviation

Levodopa

Oxidised chitosan

Levodopa/Oxidised chitosan

Levodopa/Oxidised chitosan/PLGA

Levodopa/Oxidised chitosan/PLGA/mof@nHA

Dopa

Ocs

DO

DOP

DOPM

Control

Control

Control

Control

Test

Table S2.

| Materials | Ra（nm） | Rq（nm） | Roughness Rmax |
| --- | --- | --- | --- |
| Dopa | 0.993 | 1.27 | 10.8 |
| Ocs | 3．74 | 5.12 | 78.6 |
| DO | 0.127 | 0.274 | 2.59 |
| DOP | 0.223 | 0.282 | 3.56 |
| DOPM | 0.230 | 0.327 | 10.3 |

AFM test mean square value results

Table S3.

Gene

BMSCs

Primers Sequence (5‘-3‘)

F: ATGCTCAGGACAGGATCAAA

**Rat**

Cell

ALP

R: CGGGACATAAGCGAGTTTCT

OCN

F: GAGGCTCTGAGAAGCATAAA

R: AGGGCAATAAGGTAGTGAA

Runx2

F: ATCATTCAGTGACACCACCA

R: GTAGGGGCTAAAGGCAAAAG

Osterix

F: GCCTACTTACCCGTCTGACTTTGC

R: CCCTCCAGTTGCCCACTATTGC

BMP-2

F: AACGAGAAAAGCGTCAAGCC

R: AGGTGCCACGATCCAGTCAT

GAPDH

F: CCTCTATGACAACACAGT

R: AGCCACCAATCCACACAG

**Mouse**

Gene

Primers Sequence (5‘-3‘)

Cell

RAW264.7

IL-1β

F: TGGAGAGTGTGGATCCCAAG

R: GGTGCTGATGTACCAGTTGG

IL-6

F: ATAGTCCTTCCTACCCCAATTTCC

R: GATGAATTGGATGGTCTTGGTCC

IL-10

F: CCCTTTGCTATGGTGTCCT

R: GTGGCCAGTTTGTTATTTAT

iNOS

F: CACCAAGCTGAACTTGAGCG

R: CGTGGCTTTGGGCTCCTC

Arg-1

F: TGTGTCCAGGCTCCAAATATAG

R: AGCAGGTAGCTGAAGGTCTC

β-actin

F: GTGACGTTGACATCCGTAAAGA

R: GTAACAGTCCGCCTAGAAGCAC

Primers for RT-PCR for osteogenesis and anti-inflammation
